# Sperm whale demographics in the Gulf of Alaska and Bering Sea/Aleutian Islands: An overlooked female habitat

**DOI:** 10.1371/journal.pone.0285068

**Published:** 2024-07-03

**Authors:** Natalie Posdaljian, Alba Solsona-Berga, John A. Hildebrand, Caroline Soderstjerna, Sean M. Wiggins, Kieran Lenssen, Simone Baumann-Pickering

**Affiliations:** Scripps Institution of Oceanography, University of California San Diego, La Jolla, California, United States of America; University of Saint Andrews, UNITED KINGDOM

## Abstract

Sperm whales exhibit sexual dimorphism and sex-specific latitudinal segregation. Females and their young form social groups and are usually found in temperate and tropical latitudes, while males forage at higher latitudes. Historical whaling data and rare sightings of social groups in high latitude regions of the North Pacific, such as the Gulf of Alaska (GOA) and Bering Sea/Aleutian Islands (BSAI), suggest a more complex distribution than previously understood. Sperm whales are the most sighted and recorded cetacean in marine mammal surveys in these regions but capturing their demographic composition and habitat use has proven challenging. This study detects sperm whale presence using passive acoustic data from seven sites in the GOA and BSAI from 2010 to 2019. Differences in click characteristics between males and females (i.e., inter-click and inter-pulse interval) was used as a proxy for animal size/sex to derive time series of animal detections. Generalized additive models with generalized estimation equations demonstrate how spatiotemporal patterns differ between the sexes. Social groups were present at all recording sites with the largest relative proportion at two seamount sites in the GOA and an island site in the BSAI. We found that the seasonal patterns of presence varied for the sexes and between the sites. Male presence was highest in the summer and lowest in the winter, conversely, social group peak presence was in the winter for the BSAI and in the spring for the GOA region, with the lowest presence in the summer months. This study demonstrates that social groups are not restricted to lower latitudes and capture their present-day habitat use in the North Pacific. It highlights that sperm whale distribution is more complex than accounted for in management protocol and underscores the need for improved understanding of sperm whale demographic composition to better understand the impacts of increasing anthropogenic threats, particularly climate change.

## Introduction

Male and female sperm whales are sexually dimorphic and the sexes have differences in behavior and habitat preference that result in differences in their distribution and seasonality [1–3]. Females and their dependent young form social groups and are known to inhabit temperate and tropical latitudes [2,4]. As males mature, they leave their social group and travel to higher latitudes, where they form bachelor groups as juveniles and are mostly solitary as they mature sexually [1,2,4]. The males are thought to make periodic migrations to lower latitude breeding grounds once they are sexually mature [1,2,4]. Recognizing these demographic differences, sperm whales are managed within the North Pacific stock by the NOAA National Marine Fisheries Service as a single demographic group [5] consisting of only adult males [6].

In the North Pacific, particularly in the Gulf of Alaska (GOA) and Bering Sea/Aleutian Islands (BSAI) regions, most sperm whale distribution data come from a combination of historical whaling data and visual surveys. Social groups were reported in whaling data in the North Pacific as far north as 50°N in the summer [7,8] with several records of sperm whales of both sexes overwintering in the western Aleutians [8–14]. Estimates for female sperm whale catches range from 6% of total catch above 50°N [8] to 80% in the western Aleutians, western Bering Sea, and the USSR defined Gulf of Alaska [12]. More recent surveys reported a sighting of a group of females and immature sperm whales in the Central Aleutians [11] and a group of eleven mixed-sex individuals, including one calf in the summer off the continental slope southwest of Kodiak Island [15]. This historical and contemporary evidence demonstrates that social groups are not restricted to temperate and tropical latitudes and that their distribution is more complex than currently represented in management assessments.

Sperm whales are deep-diving cetaceans that spend more than 70% of their time in foraging dive cycles [16]. The high proportion of time spent at depth makes them difficult to study using typical visual line-transect surveys, but they are excellent candidates for Passive Acoustic Monitoring (PAM) due to their high-amplitude and easily identifiable echolocation signals [17]. Three acoustic studies have documented the presence of sperm whales in the GOA [18–20]. Additional recordings from more sites with longer time series would allow for characterization of the spatiotemporal patterns of these animals.

Differences between male and female body size is linked to differences in sperm whale click characteristics [21–23]. Sperm whales produce broadband echolocation clicks in the 8 Hz to 20 kHz band, with a distinct spectral shape and a peak frequency at about 10 kHz [24]. Sperm whale echolocation clicks have a multipulse structure [25], and the time between these pulses is called the Inter-Pulse Interval (IPI). The IPI is a result of the time taken for the click to reflect multiple times between air sacs at opposite ends of the spermaceti organ and to exit the rostrum in several subsequent pulses [24,26]. Since the length of the spermaceti organ, or the rostrum of the animal, is about one-third of the total body length [27], stereo photogrammetry measurements of body length and the speed of sound in the spermaceti organ allow for the derivation of two equations (based on two different populations) that relate IPI measurements to body length [21,22]. Several studies have used manual and automatic extraction methods to estimate the acoustic length from IPIs recorded by acoustic tags and single sensor instruments [28,29]. Average IPI values range from 2–9 ms which translates to an acoustic body length estimate of 7.7 to 17.8 m. A key application of these studies is to differentiate male and female animals based on their IPI and inferred body size.

Due to source directionality, most recorded sperm whale clicks do not display a clear multipulse structure and tend to have complex pulse trains [29–31]. This limitation results in sparse information about demographic composition since the number of IPI measurements that are possible from acoustic recordings is limited. An alternative approach is to use the Inter-Click Interval (ICI), or the time between pulse trains, as a proxy for sperm whale body size and sex, particularly for large-scale acoustic monitoring where clicking bouts can last for hours [23]. Adult males and females also have different ICIs, with males clicking every ~1000 ms and females clicking every ~500 ms [32], which is like other odontocetes that display a relationship between ICI and body size [33].

In this study, we used acoustic recordings of sperm whale echolocation clicks and the differences in their click characteristics (e.g., inter-pulse interval and inter-click interval) to derive spatiotemporal patterns for male and female sperm whales at five sites in the GOA and two sites in the BSAI spanning the years 2010 to 2019. These data were investigated on an hourly and daily level to understand temporal and spatial habitat use. Generalized additive models (GAMs) with generalized estimation equations (GEEs) were used to evaluate significant spatiotemporal patterns for males and females and compared to available literature, including historical whaling data. Additionally, we used Generalized Linear Models (GLMs) to explain the relationship between sperm whale presence and drivers of presence like small- and large-scale climate variability. This study provides a baseline for sperm whale demographic presence and builds on spatiotemporal patterns that can be used as a future comparison for a region experiencing environmental change. The demographic complexities revealed in this study suggest the need to re-evaluate management of the North Pacific stock, which currently only accounts for adult male presence. Demographic specific responses to climate change should be accounted for to develop the most effective plans for conservation and protection of this species.

## Methods

### Data collection

**Fig 1:**
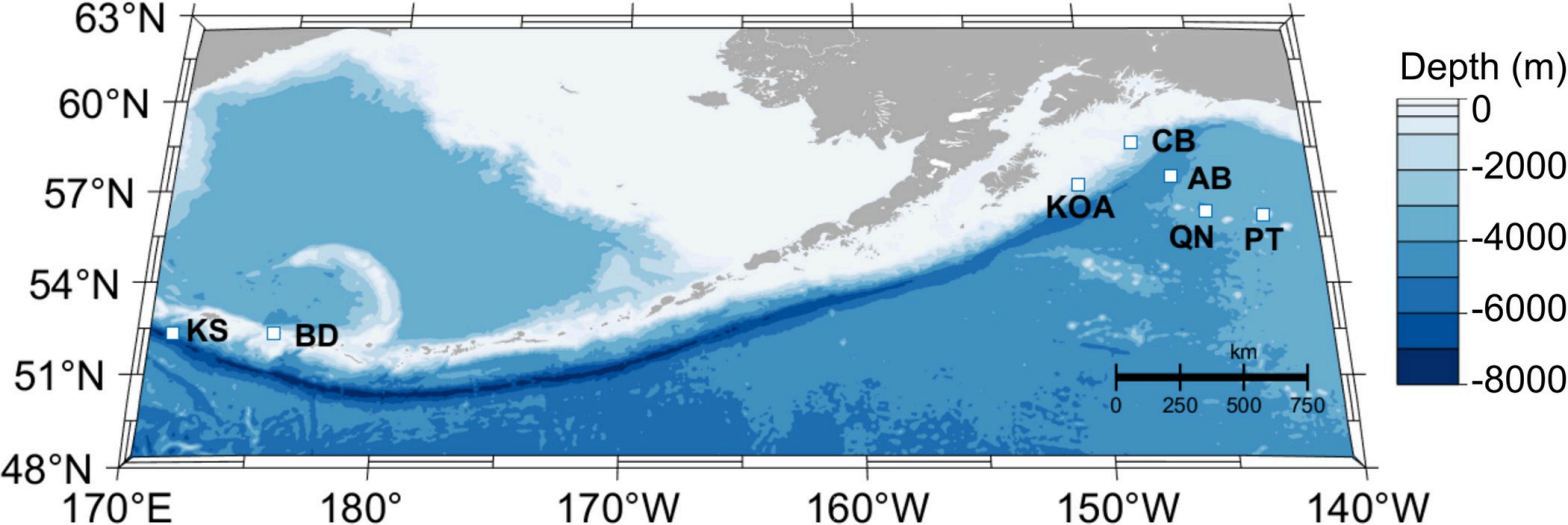
Site map. Recording locations (square markers with site abbreviations) in the GOA and BSAI regions. Bathymetry represented by blue color scale in meters.

High-frequency Acoustic Recording Packages (HARPs; 34) collected passive acoustic recordings at seven sites, two along the BSAI and five in the GOA, between June 2010 and September 2019 (Fig 1 and Table 1). For a detailed understanding of the design and deployment of HARPs, refer to Wiggins & Hildebrand (2007) [34], as only a concise description of these autonomous seafloor-mounted acoustic recorders is given here for clarity. Each site had one to ten deployments spanning a few months to five years of recording at each site. Individual site temporal coverage varied due to project goals, recorder battery life, data storage space, and duty cycle regimes (Table 1). The HARPs had a sampling rate of 200 kHz which can detect the high-frequency echolocation clicks of odontocetes, including but not limited to sperm whales. The instruments sat 10 m above the seafloor and were in moderate water depths of 780 m to 1200 m (Table 1). No permits were required for this research as the study sites were outside of marine sanctuaries, with no direct interactions with vertebrate species.

**Table 1:** Summary of recording effort.

Region	Site	Latitude (N)	Longitude (W)	Depth (m)	Recording Dates (MM/DD/YY)	No. of Recording Days
Gulf of Alaska (GOA)	Continental Slope (CB)	58° 38.74’ 58° 40.28’ 58° 40.41’ 58° 40.31’ 58° 40.26’ 58° 40.25’ 58° 39.32’ 58° 40.26’ 58° 40.22’ 58° 40.18’	148° 04.13’ 148° 01.25’ 148° 00.55’ 148° 01.31’ 148° 01.46’ 148° 01.46’ 148° 05.48’ 148° 01.45’ 148° 01.62’ 148° 01.57’	1000 900 877 858 914 900 929 874 900 972	07/13/11–02/19/12 05/03/12–02/21/13* 06/06/13–09/05/13 09/05/13–04/28/14 04/29/14–09/09/14 09/09/14–05/02/15 05/01/15–09/06/15 04/30/17–09/12/17 09/14/17–06/16/18 04/25/19–09/27/19	221 294 91 235 133 235 128 135 275 155 **1902**
				
	Pratt Seamount (PT)	56° 14.61’ 56° 14.64’ 56° 14.58’ 56° 14.60’	142° 45.44’ 142° 45.43’ 142° 45.41’ 142° 45.46’	989 987 988 987	09/09/12–06/10/13 06/11/13–08/20/13 09/03/13–03/21/14 04/30/14–09/10/14	275 70 199 134 **678**
				
	Quinn Seamount (QN)	56° 20.36’ 56° 20.48’ 56° 20.44’ 56° 20.48’	145° 11.24’ 145° 10.99’ 145° 11.11’ 145° 10.99’	930 900 994 964	09/11/13–04/16/14 09/10/14–05/02/15 05/02/15–08/18/15 04/30/17–09/14/17	217 234 109 138 **698**
				
	Abyssal Deep (AB)	57° 30.82’	146° 30.05’	1200	04/28/17–09/14/17	**139**
					
	Kodiak Island (KOA)	57° 13.44’	150° 31.70’	1000	04/24/19–09/27/19	**157**
Bering Sea and Aleutian Islands (BSAI)	Buldir (BD)	52° 38.00’ 52° 04.56’	175° 37.99’ 175° 38.39’	783 777	08/27/10–05/26/11 05/31/11–08/11/11*	272 438 **712**
				
	Kiska (KS)	52° 19.01’	178° 31.24’	1092	06/03/10–08/26/10	**84**

Summary of recording effort in the GOA and BSAI regions from 2010 to 2019. Each row represents an individual deployment. Recording effort includes region, site name (abbreviation), latitude, longitude, depth, recording dates, and total number of recording days for each deployment with the site total bolded in the final row. Deployments marked with an asterisk (*) have a duty cycle: The second continental slope (CB) deployment had a 10-minute recording duration every 12 minutes and the second Buldir Island (BD) deployment had a 5-minute recording duration every 10 minutes. The instrument locations varied slightly because of the challenges associated with deploying seafloor moorings.

### Detecting sperm whales

Sperm whale echolocation clicks were detected using the multi-step approach described in Solsona-Berga *et al*. 2020 (appendix) [35]. These clicks have multiple pulses [25], 2–9 ms apart, depending upon the size of the animal [26]. As a result, the detector had a lockout for clicks separated by less than 30 ms to avoid multiple detections of a single click. Band-pass filtering the data (5–95 kHz) minimized the effects of low-frequency noise from vessels, weather, or instrument self-noise on detections, but allowed for detection of the echolocation clicks of toothed whales. The Power Spectral Density (PSD) of detected signals was calculated with the *Pwelch* method (MATLAB, 36) using 4 ms of the waveform and a 512-point Hann window with 50% overlap [34]. Instrument specific full-system transfer functions were applied to account for the hydrophone sensor response, signal conditioning electronics, and analog-to-digital conversion. To provide a consistent detection threshold, only clicks exceeding peak-to-peak (pp) sound pressure level (RL) of at least 125 dBpp re 1 μPa were analyzed. This threshold was chosen to eliminate noise signals and the echolocation clicks of other odontocetes, while retaining sperm whale clicks.

Sperm whale echolocation clicks can be confused with the impulsive signals from ship propeller cavitation. An automated classifier developed by Solsona-Berga *et al*. 2020 (appendix) [35] was used to exclude periods of ship passages. The classifier identified potential ship passages from long-term spectral averages (LTSA), which are long duration spectrograms [37]. Further averaging was calculated as Average Power Spectral Densities (APSD) per 2-hour blocks over low (1–5 kHz), medium (5–10 kHz), and high (10–50 kHz) frequency bands with 100 Hz bins and 50% overlap. Using received sound levels, transient ship passage signals were separated from odontocete echolocation clicks and weather events. A trained analyst manually reviewed identified ship passages using the MATLAB-based custom software program *Triton* [37]. Ship passage times were removed from further analysis and considered time periods with no effort.

Instrument self-noise and the echolocation clicks of other odontocetes were also removed to reduce the number of false positive detections. A classifier using spectral click shape was implemented, taking advantage of a sperm whale click’s distinct low-frequency spectral shape to remove dissimilar clicks by delphinid and beaked whales, which typically have higher frequencies [35]. The remaining acoustic encounters containing putative sperm whale echolocation clicks were manually reviewed with *DetEdit*, a custom, MATLAB-based graphical user interface (GUI) software program used to view, evaluate, and edit automatic detections [35].

### Click characteristics as a proxy for demographics

**Fig 2:**
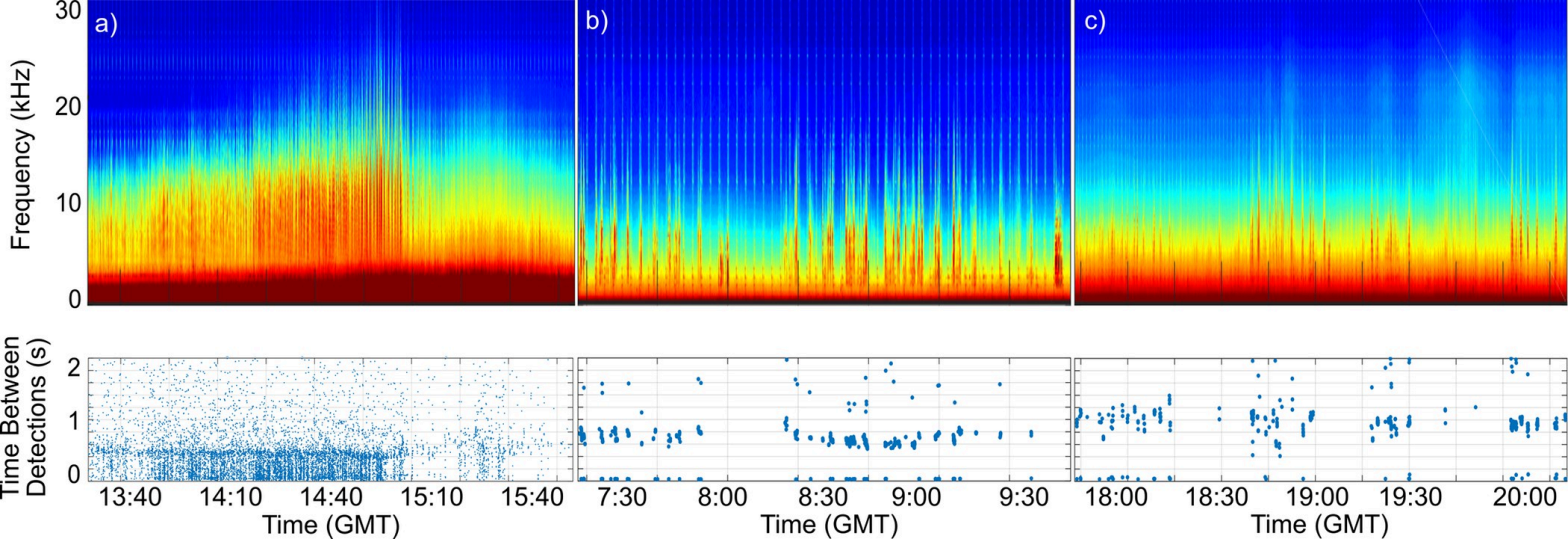
Visualization of sperm whale echolocation clicks. Sperm whale echolocation clicks in long-term spectral average (LTSA; top panel) with their time between detections (ICI; bottom panel). The panels represent three different modal ICIs: a) 500 ms s, b) 700 ms, and c) 1000 ms. The size of the points on the first panel (a) were minimized for ease of visualization of the modal ICI.

Histograms of ICI provide a visualization that can be used to indicate sperm whale size and sex [23]. A plot of concatenated histograms, referred to as ICIgrams, was annotated and categorized for each time period at each site. Examples of the ICIgram GUI can be found in Solsona-Berga *et al*. (2022) [23]. We used three ICI groups to correspond to three size classes (Fig 2, bottom panels), as per Solsona-Berga *et al*. (2022) [23]. Detections with a modal ICI of 600 ms or less were presumed to be females and their young, hereinafter referred to as Social Groups. Detections with a modal ICI of 800 ms and greater were presumed to be adult males, hereinafter referred to as Adult Males. The detections with a modal ICI between the Social Groups and Adult Males (< 600 ms and > 800 ms) could contain large females or juvenile males, hereinafter referred to as Mid-Size.

The ICIgram method was originally developed for sperm whales in the Gulf of Mexico [23], where the population consists of mostly female/immature whales and is known to have smaller body size (median difference = 1.4 m) compared to the same demographic in the Gulf of California [38,39]. To compare how effectively the ICIgram method can be used to categorize the size/sex of sperm whales in the GOA/BSAI, length estimates using IPI from individual animals were matched with the size/sex classification using the ICIgram method.

IPIs were extracted using the Cachalot Automatic Body Length Estimator (CABLE) of Beslin *et al*. (2018) [32]. This tool estimates the body length of sperm whales by compiling and clustering their IPI distributions. To avoid including the same animal more than once, only unique IPI values, or values (up to four significant figures) that were not duplicates, were retained in the final analysis. The length of the whale was estimated using two equations derived from regression analysis of IPI measurements and photogrammetrically estimated body lengths. The Gordon (1991) equation was developed based on measurements from eleven sperm whales off Sri Lanka and was applied to animals less than 11 m in length [28]. The Growcott *et al*. (2011) [22] equation was developed based on measurements from 33 large male sperm whales off Kaikoura, New Zealand and was applied to animals greater than 11 m in length [28].

### Click detection binning

Sperm whale click detections were binned into 5-minute intervals. The mean daily presence per week was calculated by summing the number of 5-minute bins with detections for each size class and for each site. Since not all sperm whale clicks were categorized into a size class, a time series of unclassified clicks was also included for each site. The ratio of hourly and daily presence for each size class was calculated and displayed with Venn diagrams to show the overlap of the classes at each site. Finally, these data were grouped into one-hour bins for statistical modeling, as described in the next section. The one-hour bins were chosen as a compromise to maintain data granularity while ensuring at least 30 minutes of recording effort in each one-hour bin for the two duty-cycled deployments.

### Statistical modeling

Generalized Additive Models (GAMs; 40) combined with Generalized Estimating Equations (GEEs; 41) were used as a model framework, outlined by Pirotta *et al*. (2011) [42], in the software *R* [43] to test the significance of temporal predictors on sperm whale presence. Patterns were explored for all sperm whales combined, hereinafter referred to as the Inclusive model, and for each of the three size classes, referenced as the Social Group, Mid-Size, and Adult Male models. Models were built for each of these groups for each site with more than 270 days of recording (BD, CB, PT, and QN), for each region (GOA and BSAI), and for an All-Site model. The response variable was binomial presence-absence of sperm whale clicks in one-hour bins (1 = presence and 0 = absence). The explanatory variable *Julian day* was included for all site-specific models while the variable *year* was only included at CB where more than five years of data were available. The region-specific models included *Julian day* and *site* (BD, KS, CB, PT, QN, AB, KOA) as explanatory variables. *Year* was only included in the regional GOA model where more than five years of data were available. Finally, for the All-Site model, *Julian day*, *region* (GOA and BSAI), and *year* were included. The variable *time lost* was originally included as the number of missing 5-minute recording bins in each hour to account for the differences in recording effort due to ship passage exclusions but ultimately removed from final models due to a lack of significance.

Sperm whale encounters lasted for many hours to days at all sites, indicating temporal autocorrelation whereby detections in a single one-hour bin increased the likelihood of detections in adjacent bins. To minimize the impacts of the temporal autocorrelation and to avoid data sub-sampling or using a coarse analysis resolution, the GAMs were combined with GEEs, a method previously used to address autocorrelation in marine mammal presence data [41,42,44,45]. Under this approach, the data are grouped into blocks, within which residuals are allowed to be correlated, while independence is assumed between separate blocks. The *R* correlation function *acf* within the *stats* package [43] was used to determine the time step for blocking. Blocks were defined by the value where the autocorrelation of the residuals of a Generalized Linear Model (GLM) dropped below 0.1. Block sizes varied between 226–1249 hours (9–52 days) for all 28 models. Although GEEs are considered robust against correlation structure misclassification [46], an autoregressive order 1 (AR-1) covariance structure was used to describe model error given the temporal autocorrelation in the data [41,42,45,47–49].

The same GLM used to determine block size was also used to assess collinearity of covariates following [50]. The *vif* (Variance Inflation Factor) function in the *R* package *car* [51] identified potentially collinear covariates. None of the variables in the GLM model had a VIF value over 2.0 and all variables were retained for further modeling.

Models were built using the function *geeglm* in the *geepack* library [52] in *R*. Variables were treated differently (spline vs. factor) within each model based on the nature of the covariate. Given the long time series at each site and region, *Julian day* was included as a cyclic spline based on a variance-covariance matrix built using the *gam* function in the *mgcv* package in *R* [53] to fit a circular smooth in a GEE framework. Given the small number of years for the time series, *year* was included as a factor to estimate year specific effects. *Site* and *region* were input into models as factors given the categorical nature of both variables.

For models with more than one variable, model selection used the Quasilikelihood under Independence model Criterion (QIC_a_) value, an alternative to Akaike’s Information Criterion for GEE models [54], available through the function *QIC* in the *geepack* library in R (v.1.1–6; 58). Manual backwards stepwise model selection was carried out where the model with the lowest QIC_a_ from the full model (all variables) and a series of models containing all terms but one, was used in the following step [42,44]. This selection method continued until removing any of the remaining covariates caused the QIC_a_ to increase. The order of the variables in the final model was determined by which variable, when removed, increased the QIC_a_ the most. A Wald’s Test was conducted on the final model using the function *anova* in the *geepack* library to access the significance of each variable in the model. Any non-significant covariates were removed from the models using backwards stepwise model selection until all p-values of the remaining covariates were less than 0.05. Partial-fit plots for each variable in the final models were created using the approach of Pirotta *et al*. (2011) [42]. The x-axis for *Julian Day* is represented by the months of the year and interpreted as sperm whale occurrence among seasons (winter: December—February, spring: March—May, summer: June–August, fall: September–November).

Goodness of fit for the models was evaluated using the *performance* package in *R* [55]. The coefficient of discrimination, also known as Tjur’s R2 [56], was calculated for each model using the function *r2_tjur*. Binned residuals were also used to assess the fit of the models. Binned residual plots were obtained using the function *binnedplot* [57]. A good fit was expected to have residuals within the 95% confidence intervals [57].

Generalized Linear Models (GLMs) examined the relationship between sperm whale presence and the El Niño Southern Oscillation’s (ENSO) via the Oceanic Niño Index (ONI), the Pacific Decadal Oscillation (PDO) index, the North Pacific Gyre Oscillation (NPGO) index, and the Marine Heatwave Watch (MHW). The monthly PDO, ONI and NPGO values were extracted using the *rsoi* package in *R* [58] and the MHW forecast was generated using Jacox *et al*. (2022) [59]. Hourly binary presence of sperm whales was averaged for each month and divided by the recording effort. To remove seasonality, the timeseries was deseasoned using the functions *stl* and *seasadj* in the *forecast* package in *R* [60,61]. Previous studies in the GOA found an 8–12 month lag between ENSO events and sperm whale peak presence [62]. And since PDO, ONI, and NPGO are connected to one another [63], 8–12-month lags were tested for these indices as well.

## Results

### Comparison of IPI and ICI

**Fig 3:**
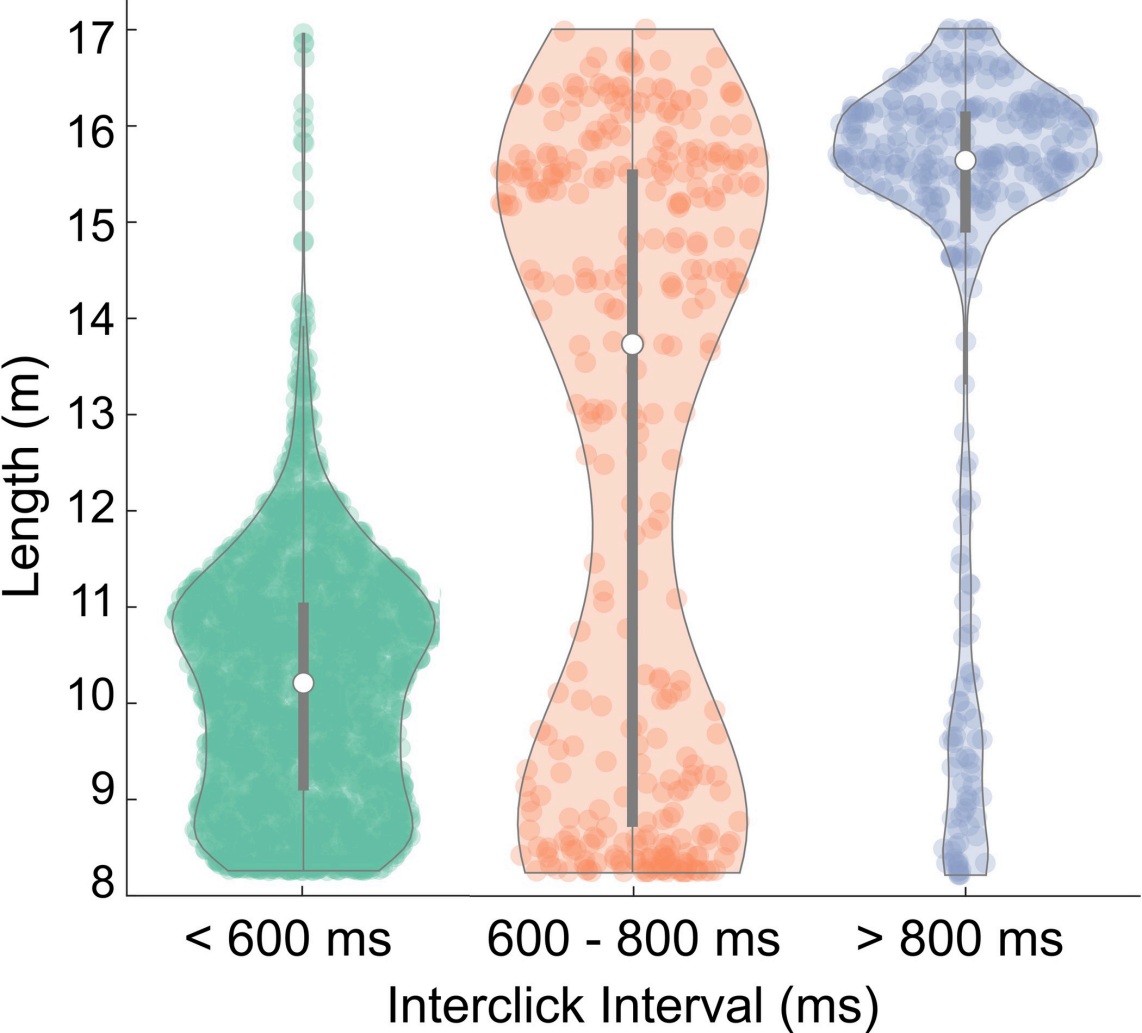
IPI vs. ICI comparison. Estimated body length (m) from the IPI categorized into the three ICI groups (< 600 ms, 600–800 ms, and > 800 ms). Median represented by the white dot and the interquartile range by the gray bar.

Sperm whale body length estimates were calculated using both their IPI and ICI for 3,047 animals identified based on a unique IPI that was not repeated and indicated an individual animal. An effort was made to extract animal lengths from acoustic data that encompassed sites with more than one year of recording (CB, BD, QN, PT), multiple seasons, and various years to account for variability. The animal lengths obtained from the IPI were divided into three ICI groups (< 600 ms, 600–800 ms, > 800 ms) obtained from the ICIgrams and the results visualized using violin plots (Fig 3). These plots reveal clear distinctions between the ICI classes based on the body lengths measured by IPI and likely correspond to three size/sex classes, Social Groups (< 600 ms), Mid-Size (600–800 ms), and Adult Males (> 800 ms). The Social Groups class is comprised of small animals with a median length of 10.2 m (n = 2,387) and a moderate interquartile range (IQR) of 1.9 m (the range of the middle 50% of the distribution). The Adult Males class has large animals with median length of 15.7 m (n = 325) and a small IQR of 1.2 m. Whereas the Mid-Size class has median of 13.6 m (n = 335), a broad range of body lengths with an IQR of 6.6 m, and a bimodal distribution that overlapped with both Social Group and Adult Male body lengths. There were outliers within the Social Groups and Adult Males classes, where the length estimates from their IPIs indicated that the ICIgram method may have misclassified the size/sex of the animal. These usually occurred during encounters when more than one size/sex class were echolocating at the same time. Only 3% of animals classified as Social Group had body sizes larger than 12 m and were likely misclassified as Social Group. For Adult Males, 14% of classified animals had body sizes smaller than 10 m and were likely misclassified as Adult Male.

The distribution of ICI size classes varied between sites (S1 Fig). Averaged across sites, the Social Groups had a mean ICI value of 680 ms, with a range of means from 600 to 700 ms across sites; the Mid-Size had a mean value of 800 ms, ranging from 750 to 800 ms across sites; and the Adult Males had a mean value of 980 ms ranging from 850 to 1050 ms across sites (S1 Fig).

### Spatial overlap of size classes

**Fig 4:**
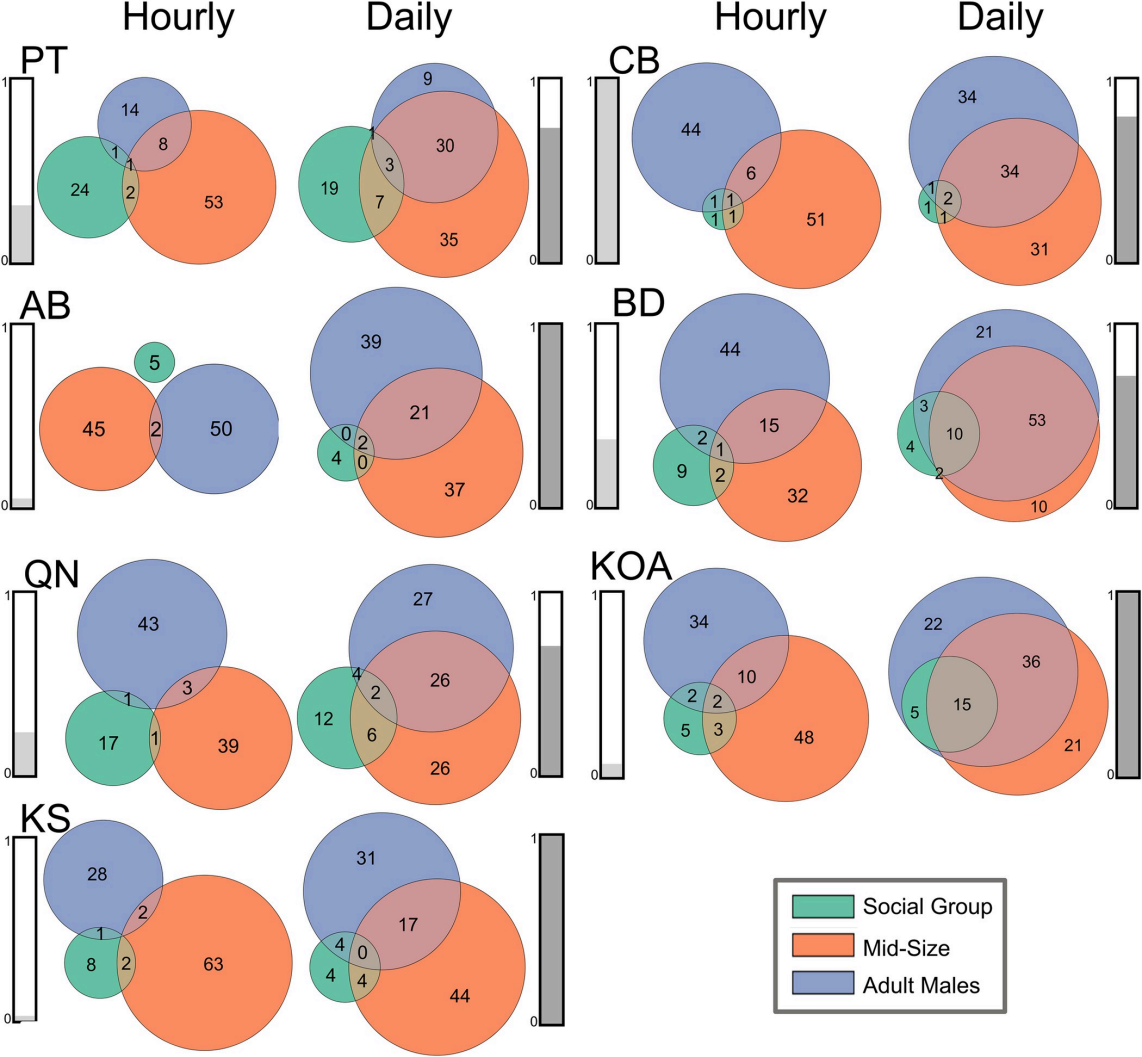
Hourly and daily presence of sperm whales. Ratio of hourly (left) and daily (right) presence of each size class at each recording site. Social Groups in green, Mid-Size in orange, and Adult Males in blue. Overlap between groups represents simultaneous presence of those groups in the same hour or day. The bars on the left of each diagram (light grey) represent normalized recording effort at that site. The bars on the right (dark grey) represent normalized sperm whale presence at that site.

All three size/sex classes were detected across all sites, with temporal overlap between classes when observed on 5-min time bin, hourly, and daily time scales (S1 Table and Fig 4). The highest proportion of overlap at all sites was between Mid-Size and Adult Males. Adult Male and Mid-Size animals were found in the same hourly bin 7% (range 2–16%) and daily bin 36% (range 17–63%) of encounters (Fig 4). Whereas for Social Groups and Mid-Size, they were found together in the same hourly bin only 2% (range 0–5%) and daily bin 8% (range 2–17%) of encounters (Fig 4). Similarly, Adult Males and Social Groups were found together in the same hourly bin 2% (range 0–4%) and daily bin 7% (range 2–20%) of encounters (Fig 4). As expected, encounters with all three size/sex classes were rare with hourly bin overlap of 1% (range 0–2%) and daily bin overlap of 5% (range 0–15%) (Fig 4).

Certain 5-min bins were ineligible for class categorization either because of a low click count or an absence of adjacent time bins to inform the categorization process. Excluding site BD, the mean percentage of 5-min bins that were not classified was 11% (range 5.1–18.5%) (S1 Table). For BD, the percentage of 5-min bins that were not classified was 33.3%, likely because nearly half of the data was duty cycled (S1 Table). Duty cycled data have gaps with no recording effort which could interfere with the requirement of the ICIgram method for a 5-min bin with detections to have neighboring time bins with detections and exacerbate the number of bins that were not classified.

At all sites, the proportion of Mid-Size and Adult Male presence was greater than Social Groups on the hourly and daily scale (Fig 4). For sites with greater than a year of recording effort, the proportion of Social Group presence in descending order by site was the two seamount sites (PT and QN), island site (BD), and the continental slope (CB) (Fig 4). For sites with less than a year of recording effort, the proportion of Social Group presence in descending order by site was the continental slope site (KOA), deepwater (AB), and island site (KS) (Fig 4). The proportion of Mid-Size and Adult Males was comparable at all sites except for PT where the proportion of Mid-Size presence was 80% larger than Adult Males (Fig 4).

### Presence by site

**Fig 5:**
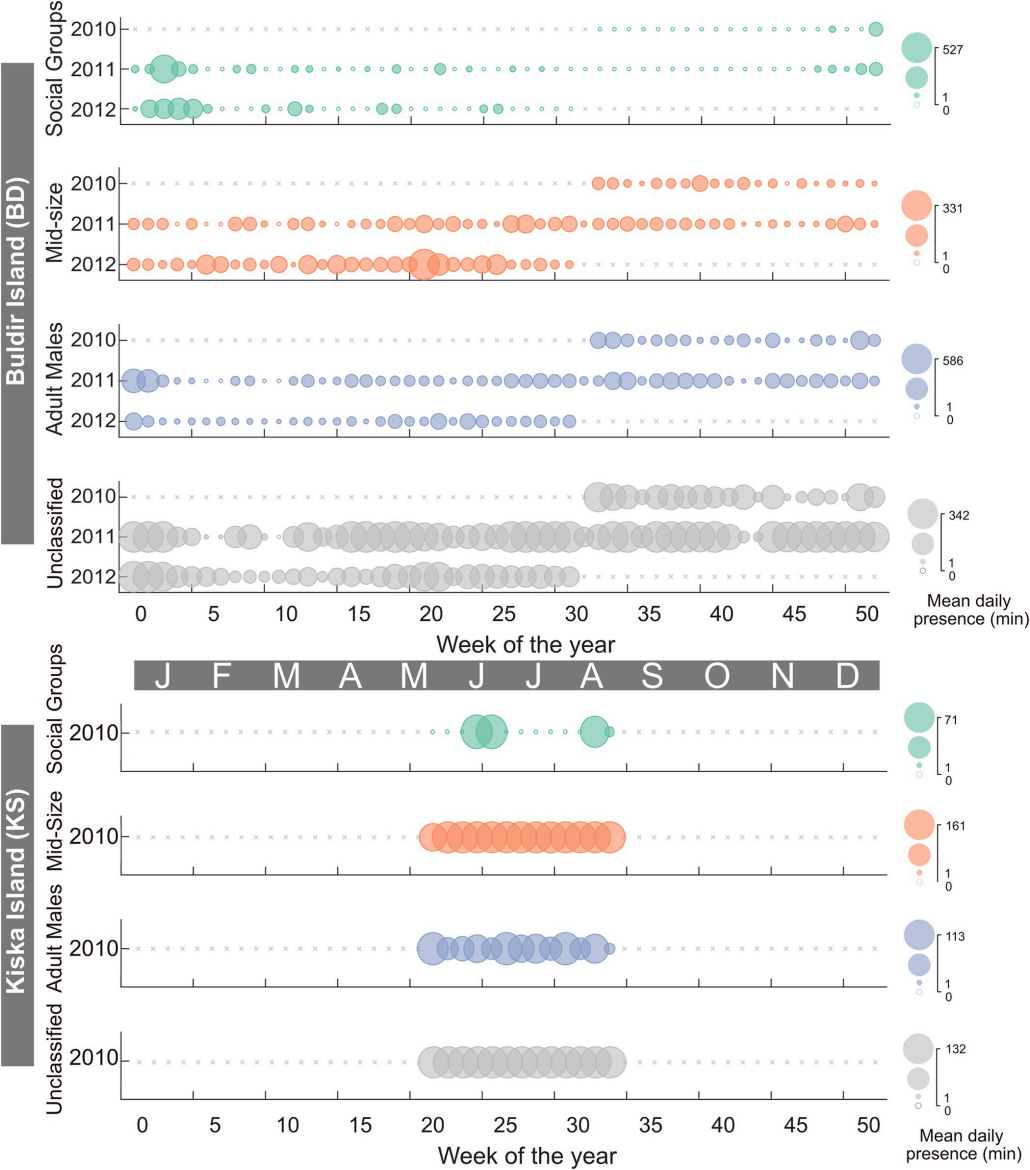
Sperm whale presence at the Buldir (BD) and Kiska (KS) Island Sites. Each row represents a year. The color of the bubble represents the size class; Social Groups by green, Mid-Size by orange, Adult Males by blue, and unidentified clicks in grey. The size of the bubble is the mean daily presence in minutes represented with a scale on the right. Grey ‘x’ symbols represent no recording effort.

**Fig 6:**
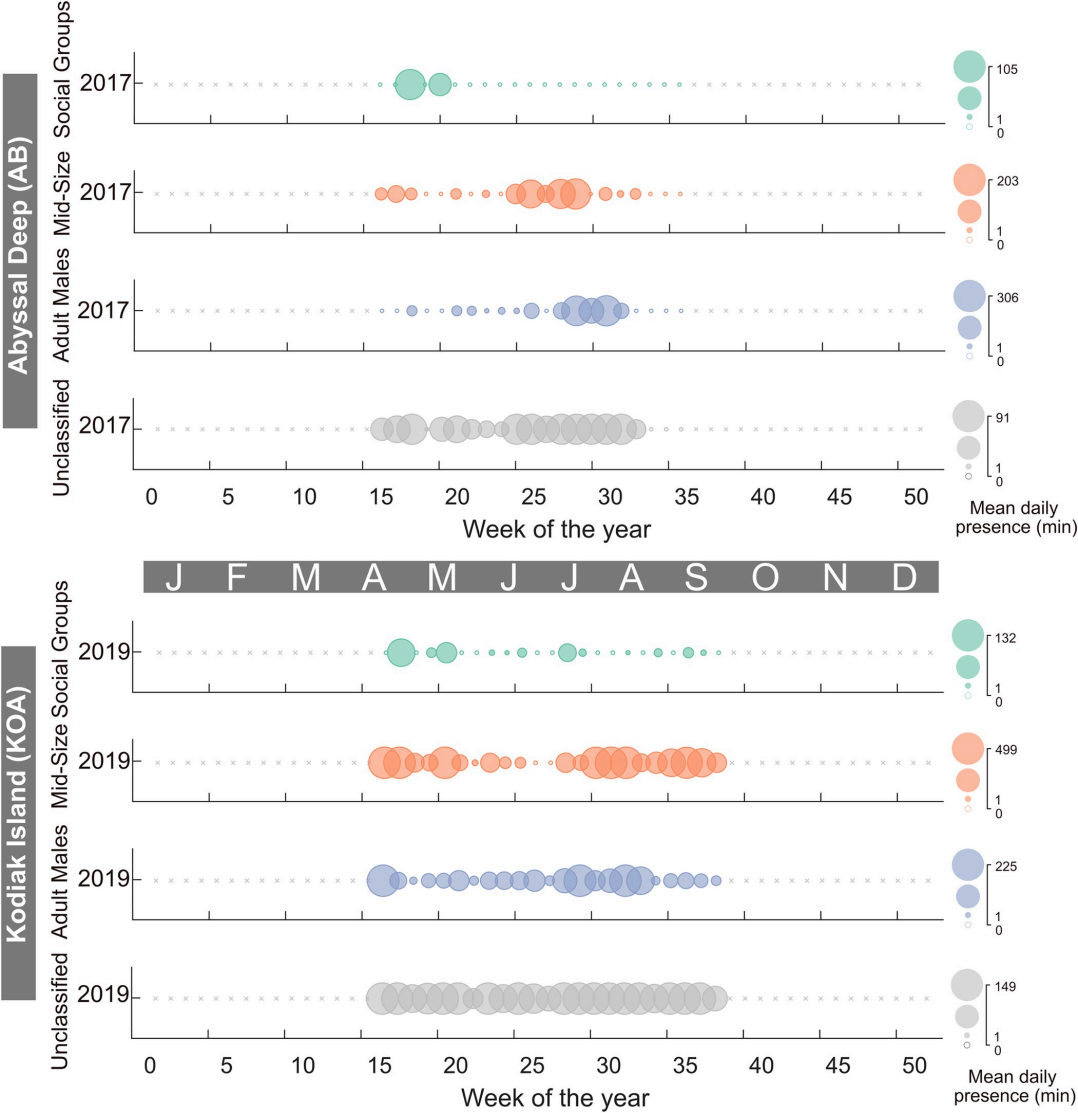
Sperm whale presence at the Abyssal Deep (AB) and Kodiak Island (KOA) sites. Colors and symbols as per Fig 5.

Sperm whales of all size/sex classes were detected at every site and presence was reported as the mean daily presence (min and hr) per week, herein after referred as daily presence. AB, KS, and KOA had the highest normalized daily sperm whale presence and the lowest normalized recording effort (Fig 4). All three sites only captured 19, 12, and 22 weeks respectively, during the spring and summer when sperm whale presence was usually the highest (Figs 5 and 6). CB had the next highest normalized daily sperm whale presence and the highest normalized recording effort (> 5 years), followed by PT, BD, and QN (Fig 4).

Sperm whales were present almost every week during the nearly two years of recording at the Buldir Island (BD) site (Fig 5). Social Groups were almost exclusively present during the winter months between 2010 and 2012 with a maximum daily presence of 527 min (8.8 hr) (Fig 5). Mid-Size were the most consistent size class present with a maximum daily presence of 331 min (5.5 hr) (Fig 5). Adult Males had a maximum daily presence of 586 min (9.7 hr) with the peak in presence seen in January of 2011 (Fig 5). Sperm whales were present every week at the Kiska Island (KS) recording site during the 13-week deployment (Fig 5). Mid-Size and Adult Male presence were higher and more consistent with a maximum daily presence 161 and 113 min (2.7 and 1.9 hr), respectively (Fig 5). Social Groups were present for 4 weeks and had a maximum daily presence of 71 min (1.2 hr) (Fig 5).

**Fig 7:**
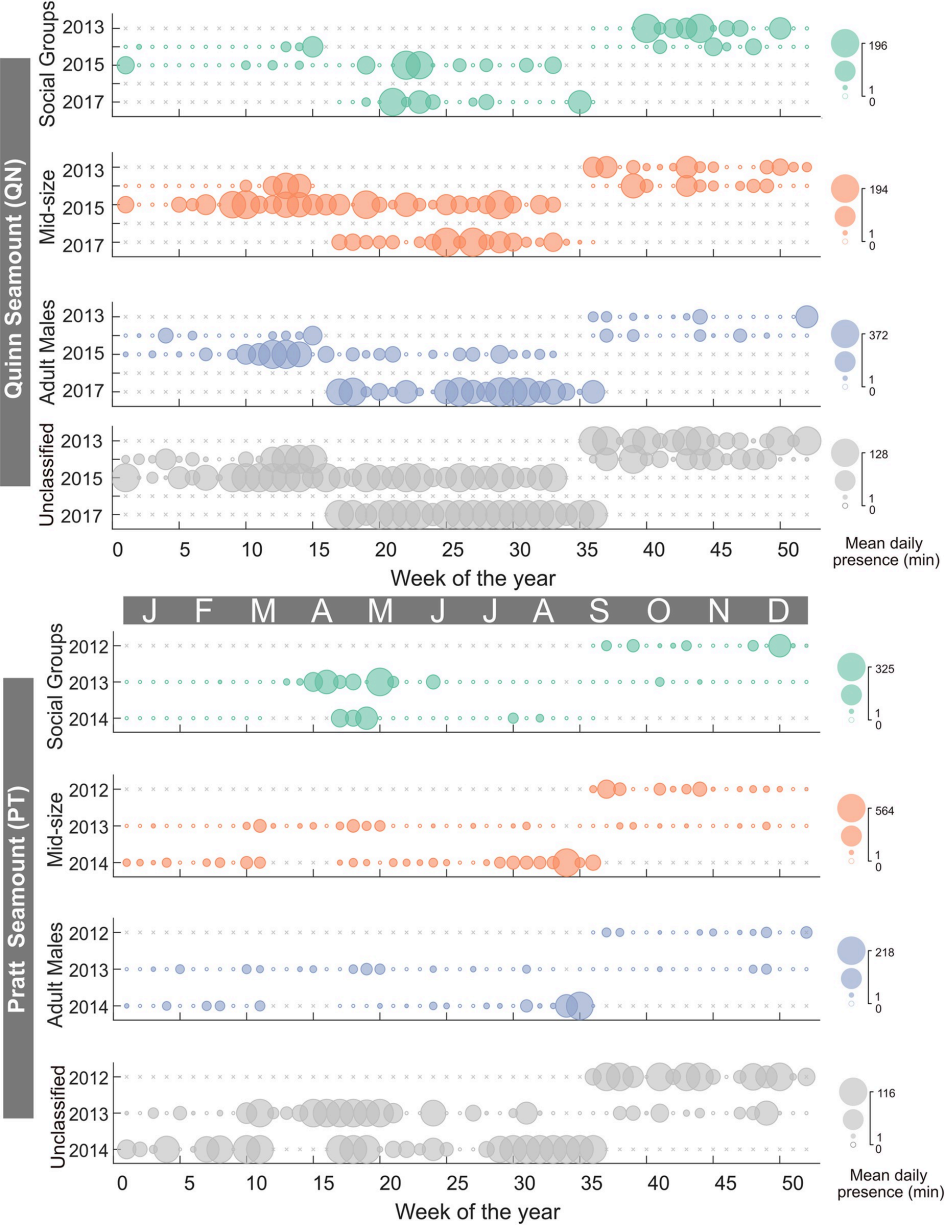
Sperm whale presence for the Continental Slope (CB) Site. Colors and symbols as per Fig 5.

**Fig 8:**
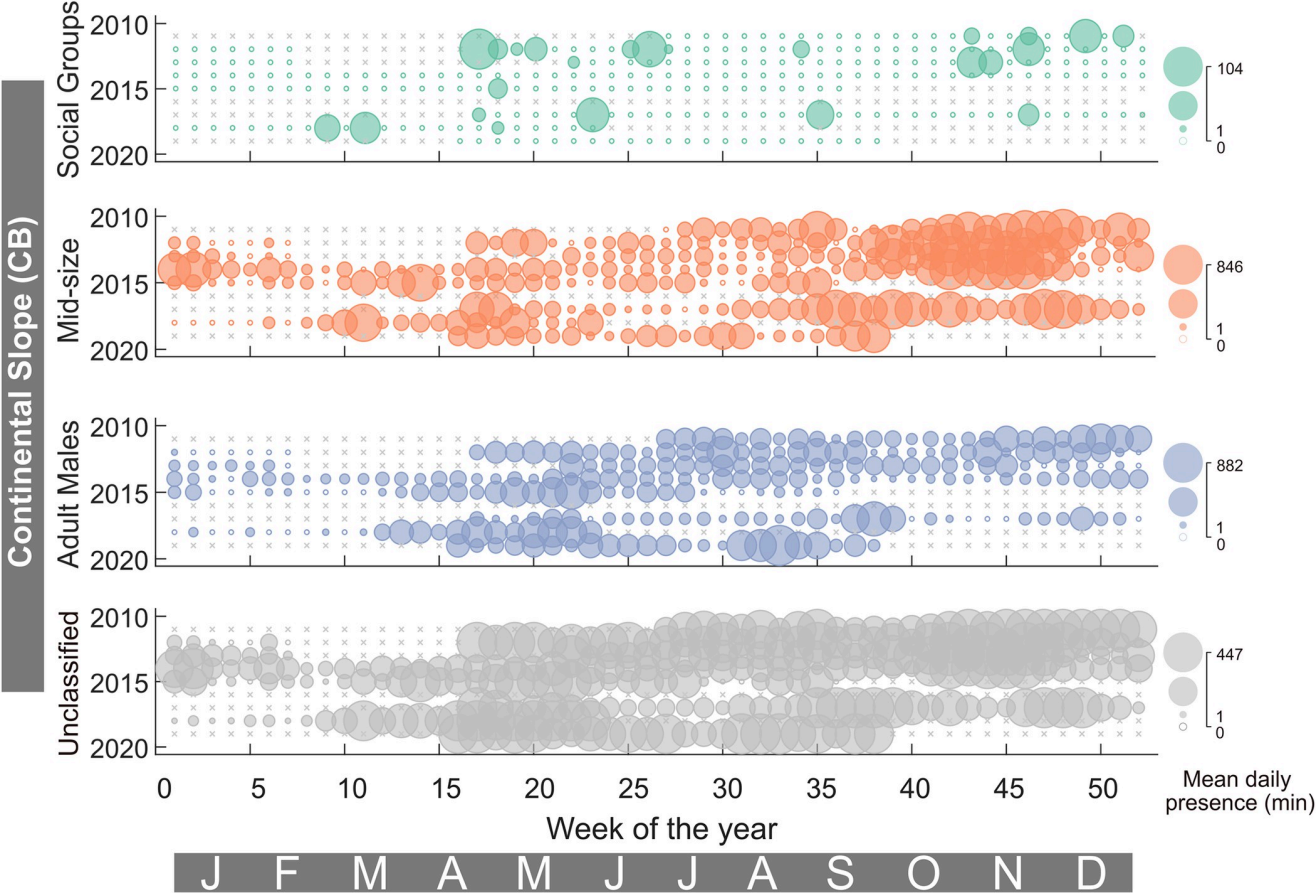
Sperm whale presence at the Quinn Seamount (QN) and Pratt Seamount (PT) Sites. Colors and symbols as per Fig 5.

In the GOA, the continental slope (CB) site had the highest level of sperm whale presence compared to the other recording sites (Fig 7). Presence at CB was dominated by Mid-Size and Adult Males with maximum daily presence of 846 and 882 min (14.1 and 14.7 hr), respectively. Social Groups were present episodically throughout the eight-year recording period with a maximum daily presence of 104 min (1.7 hr). The two seamount sites, Quinn (QN) and Pratt (PT), had a more consistent presence of all size classes throughout the recording period (Fig 8). The maximum daily presence of Social Groups, Mid-Size, and Adult Males at QN were 196, 194, and 372 min (3.3, 3.2, and 6.2 hr), respectively (Fig 8). The maximum daily presence of Social Groups, Mid-Size, and Adult Males at PT were 325, 564, and 218 min (5.4, 9.4, and 3.6 hr), respectively.

Sperm whales were present every week of the recording period for the Abyssal Deep (AB) and Kodiak Island (KOA) sites in the GOA. Compared to the other size classes, there was less Social Group presence at AB and KOA with a maximum daily presence of 105 and 132 min (1.8 and 2.2 hr), respectively (Fig 6). There was more presence of Mid-Size and Adult Males at KOA with a maximum daily presence of 499 and 225 min (8.3 and 3.8 hr), respectively (Fig 6). AB had a maximum daily presence of Mid-Size and Adult Males of 203 and 306 min (3.4 and 5.1 hr), respectively (Fig 6).

### Modeling

Sperm whale presence was modeled for all sperm whale classes included together (Inclusive model), and for each of the three size classes independently (Table 2). Data were used from selected sites with good seasonal coverage (BD, CB, PT, and QN), from all the sites in each region (GOA and BSAI), and from all the sites combined (All-Site).

**Table 2:** GEE model summaries.

Model	Site/ Region	Variable	Model Output	Inclusive	Social Groups	Mid-Size	Adult Males
Site	BD	Julian Day	P	0.00020 ***	2.8e-09 ***	7.2e-05 ***	6e-06 ***
			Df	2	2	2	2
			X^2^	16.6	39.4	19.1	24.1
	PT	Julian Day	P	0.026 *	0.00080 ***	0.016 *	NA
			Df	2	2	2	
			X^2^	7.3	14.2	8.3	
	QN	Julian Day	P	7.8e-09 ***	0.0032 **	1.1e-09 ***	6e-08 ***
			Df	2	2	2	2
			X^2^	37.3	11.5	41.3	33.3
	CB	Julian Day	P	NA	0.038^2^ *	1.2e-05 ***	3.8e-06***
			Df		2	2	2
			X^2^		6.6	22.6	24.9
		Year	P	3e-05***	<2e-16^1^ ***	NA	0.00030 ***
			Df	7	7		7
			X^2^	32.7	5437.6		27.66
Region	BSAI	Julian Day	P	0.0014 **	1.2e-06 ***	1.7e-07 ***	1.8e-05 ***
			Df	2	2	2	2
			X^2^	13.2	27.3	31.2	21.9
		Site	P	4e-05 ***	0.0066 **	--	0.00020 ***
			Df	1	1		1
			X^2^	16.9	7.4		14.4
	GOA	Year	P	<2e-16 ***	0.0031^1^ **	7.2e-08 ***	NA
			Df	7	7	7	
			X^2^	121.5	21.5	46.4	
		Julian Day	P	0.00083 ***	2.5e-05^3^ ***	4.7e-06 ***	0.012^2^ ***
			Df	2	2	2	2
			X^2^	14.2	21.4	24.5	8.8
		Site	P	NA	< 2.2e-16^2^ ***	NA	< 2e-16^1^ ***
			Df		4		4
			X^2^		8513.6		91.23
All-Site		Year	P	<2e-16 ***	0.017 *	0.00019 ***	<2e-16 ***
			Df	8	8	8	8
			X^2^	119.9	18.6	30.2	132.2
		Julian Day	P	7.04e-06 ***	7.2e-06 ***	5.1e-06 ***	8e-08 ***
			Df	2	2	2	2
			X^2^	23.7	23.7	24.4	32.7
		Region	P	NA	NA	NA	NA
			Df				
			X^2^				

Model summaries for each site, regional, and All-Site models for the Inclusive, Social Groups, Mid-Size, and Adult Male classes. Model summaries include the p-value (P), degrees of freedom (Df), and the Chi-square statistic (X^2^). The significance of the p-value is indicated by the following codes

‘***’ <0.001

‘**’ <0.01, and

‘*’ <0.05. If a model had more than one variable, the listed order of the variables represents the order they were input into the model. Models that had different input orders have a superscript for the p-value indicating the order it was input into the model. Covariates that were not retained in the model or not significant are represented with ‘NA’.

The average annual percentage of one-hour bins with presence for the Inclusive, Social Group, Mid-Size, and Adult Male models was 98% (8579), 4% (362), 44% (3902), and 32% (2779), respectively (S2 Table). The highest performing models were the Adult Male models with 32 to 50% of Residuals within the 95% confidence intervals. The lowest performing models were the Social Group models with 7 to 25% within the 95% confidence intervals (S2 Table). The models had low Tjur’s R2 values and % of Residuals within the 95% confidence intervals suggesting that the temporal (Julian day and year) and spatial (site) variables alone included in the models are not good predictors of animal detections.

### Seasonal patterns

**Fig 9:**
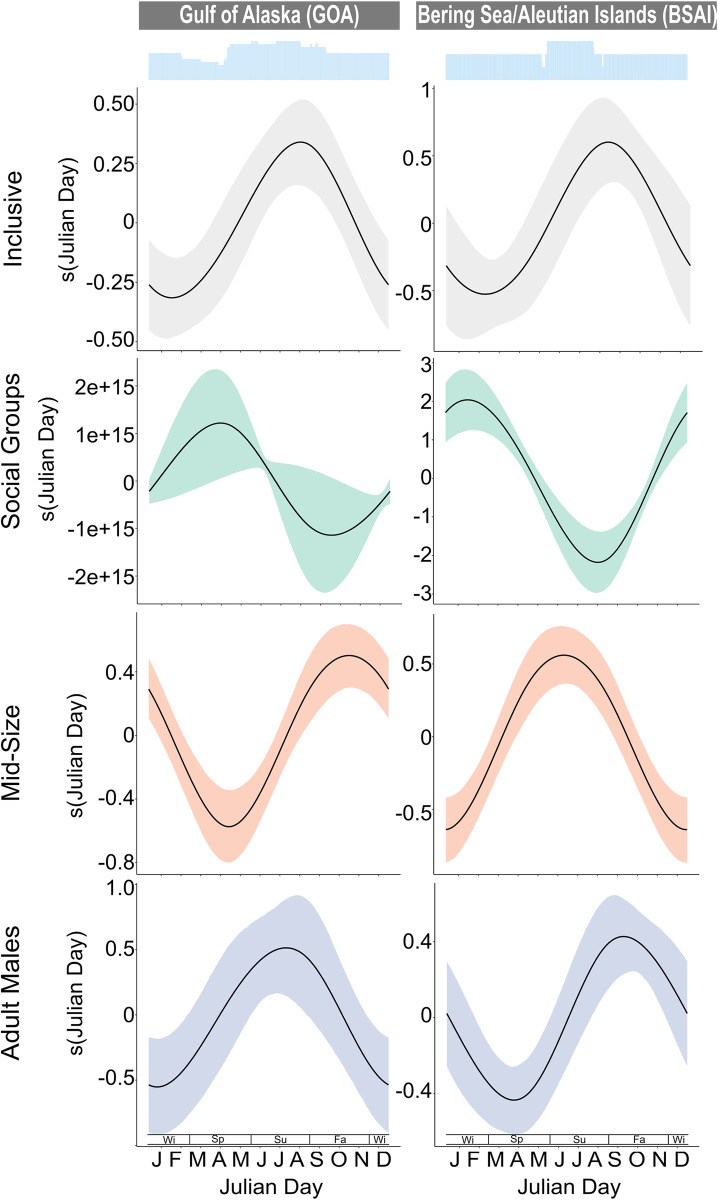
Seasonal plots for the sites in the Gulf of Alaska and the Bering Sea/Aleutian Islands. Gulf of Alaska (left) and the Bering Sea/Aleutian Islands (right) seasonal plots. Each row represents outputs from the different size class models for each site: a) Inclusive (grey), b) Social Groups (green), c) Mid-Size (orange), and d) Adult Males (blue). Julian day is represented as months as well as by seasons (Wi: December—February, Sp: March—May, Su: June–August, Fa: September–November). The blue histograms at the top denote effort. All plots include 95% confidence intervals represented by the grey shading surrounding the smooth.

Significant seasonal patterns were found in the majority (26 out of the 28) of final models (Table 2). The Inclusive models revealed a seasonal pattern of increased presence in the summer for all GOA sites and fall for the BSAI sites (Fig 9). The patterns revealed by the Inclusive models were like those of the Adult Males at all sites where presence was highest in the summer for GOA and fall for BSAI (Fig 9). The seasonal patterns for Mid-Size and Social Groups were more nuanced and varied from site to site. For the Mid-Size, peak presence was seen in the summer or fall across all sites and regions except QN where peak presence was observed in the spring (Fig 9, S2 and S3 Figs). For Social Groups, peak presence was seen in the spring, except for QN and BD where the peak in presence was in the fall and winter, respectively (Fig 9, S2 and S3 Figs). Peak presence of the Social Groups rarely overlapped with those of the Adult Males.

### Interannual variability

**Fig 10:**
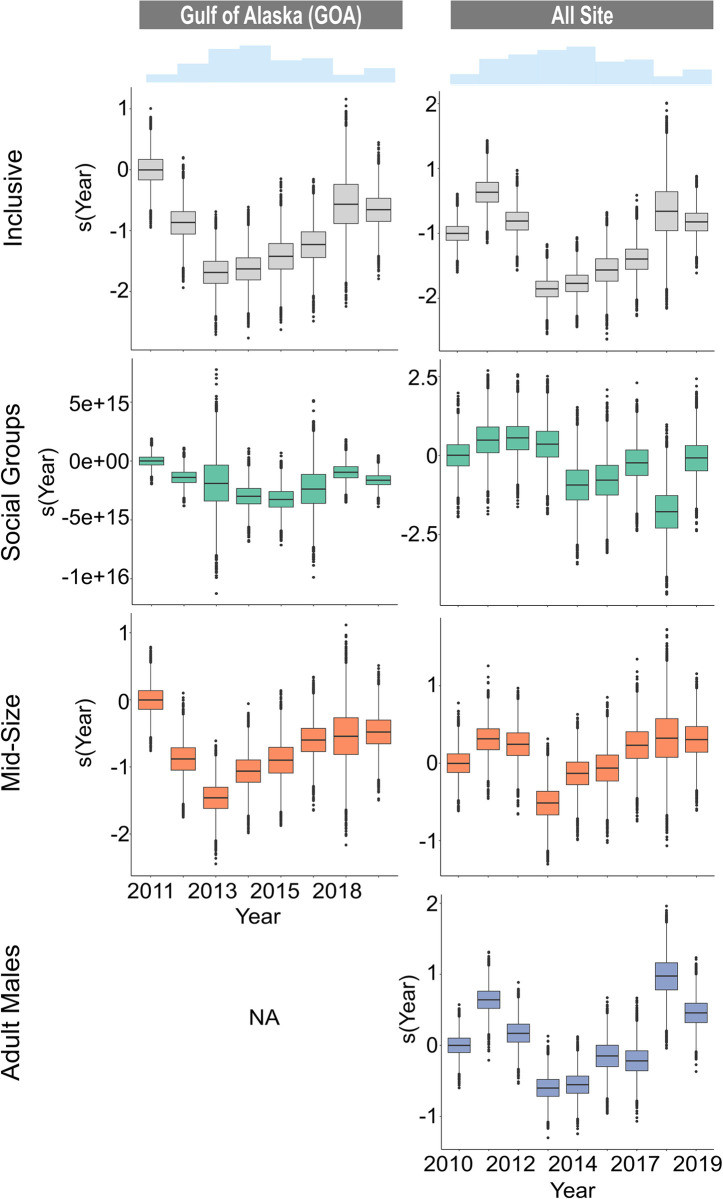
Yearly presence for the Gulf of Alaska and all sites combined. Presence by year for the Gulf of Alaska region (left) and All Sites (including Bering Sea/Aleutian Islands) (right). Each row represents outputs from the different size class models for each site: a) Inclusive (grey), b) Social Groups (green), c) Mid-Size (orange), and d) Adult Males (blue). Year is a categorical variable displayed as box plots with the first level centered on zero. Covariates that were not retained in the model or not significant are represented with ‘NA’.

Long-term, interannual variability was only assessed for the CB site, GOA region, and the All-Site models where there was more than five years of data (S3 Fig and Fig 10). At CB, the Inclusive and Adult Male models revealed a decrease in presence every year after 2011 with the lowest presence in 2014, 2015, and 2017, followed by an increase in 2018 and 2019 (S3 Fig). For Social Groups, presence remained steady from year to year except in 2014 and 2019 where there was no Social Group presence whatsoever. Interannual variability was not significant for Mid-Size at CB. In the GOA, the Inclusive and Mid-Size models revealed a decrease in presence every year after 2011 with the lowest presence in 2013 and 2014, followed by an increase every subsequent year (Fig 10). Social Group presence remained steady from year to year with the highest presence in 2011 and a small dip in presence in 2014 and 2015. Interannual variability was not significant for Adult Males in the GOA region. For all seven sites, the Inclusive, Mid-Size, and Adult Male models revealed an increase in presence in 2011, followed by a decrease in presence and a minimum in 2013 with increasing presence in subsequent years. Social Group presence remained consistent from year to year with a dip in presence starting in 2014 and the lowest presence in 2018.

### Environmental variability

**Fig 11:**
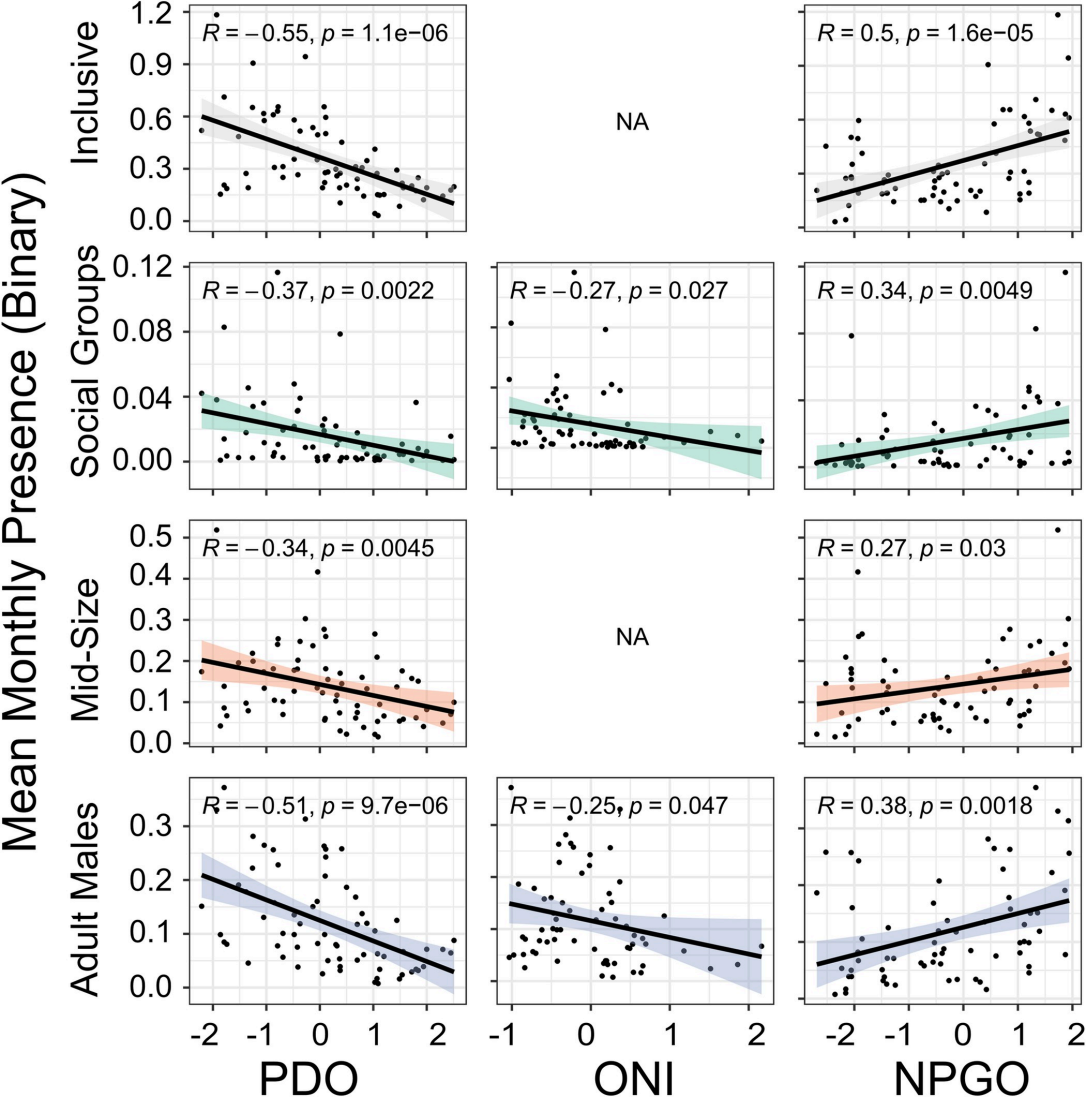
Relationship between sperm whale presence and environmental variables. GLM plots displaying the relationship between mean monthly presence of sperm whales for All-Sites and the PDO, ONI, and NPGO index. All PDO and NPGO plots represent an eight-month lag, and all ONI plots represent a nine-month lag. Each row (and color) represents outputs from the different size class models for each variable: Inclusive, Social Groups, Mid-Size, and Adult Males. All plots include 95% confidence intervals represented by the grey shading surrounding the linear regression. The regression formula for each model is displayed in the top left-hand corner. Covariates that were not retained in the model or not significant are represented with ‘NA’.

Sperm whale presence was correlated to the PDO, ONI, and NPGO indices in varying degrees depending on the model (S3 Table, Fig 11 and S6–S10 Figs). The PDO index with an eight-month lag was significant for all models except for the Mid-Size at CB and Social Groups at GOA (S3 Table). All significant models revealed a negative correlation between the PDO index and sperm whale presence (Fig 11 and S9 and S10 Figs). The decrease in sperm whale presence in 2013 aligns with the inflection point of the PDO from a cool to warm phase (S6–S8 Figs). The ONI was significant for less than half of the models with a nine-month lag being consistently significant for all models (S3 Table). All significant models revealed a negative correlation between the ONI and sperm whale presence (Fig 11 and S9 and S10 Figs). The decrease in sperm whale presence in 2013 aligns with the ENSO becoming neutral and is sustained as it transitions to El Niño (warm phase) (S6–S8 Figs). The NPGO index was significant for all models except for the Inclusive at CB, Social Groups at GOA, and all Mid-Size models (S3 Table). Like the PDO index, an eight-month lag was significant for all models except for the Social Groups at CB. All significant models revealed a positive correlation between the NPGO index and sperm whale presence (Fig 11 and S9 and S10 Figs). The decrease in sperm whale presence in 2013 aligns with the inflection point of the NPGO from a positive to a negative phase (S6–S8 Figs). R^2^ values for all the linear regressions revealed a weak correlation with values ranging between +/-0.27 and +/-0.55 (S3 Table). The MHW forecast was not significant in any of the models (S3 Table) although less sperm whale presence does appear to align with higher MHW probability (S6–S8 Figs).

## Discussion

This study analyzed demographic composition of sperm whales at seven sites in the Gulf of Alaska and Bering Sea/Aleutian Islands over a wide range of years and seasons. Three size/sex classes were identified (Social Groups, Mid-Size and Adult Males) based on their echolocation ICI, supported by examining IPI for individual clicks (Fig 3). The median body length of animals in the Social Group class (10.2 m; Fig 3) is comparable to the average body lengths documented for sperm whale females and immature animals that ranges from 8 to 11 m [1,38,64–67]. The median length of the Adult Male class (15.7 m; Fig 3) suggests that the males in this study are both physically mature [occurs at a mean length 15.5–15.9 m [68]] and sexually mature [occurs from 9.5 m [27] to 13.8 m [69]]. The Mid-Size class displayed a bimodal distribution that overlapped with the Social Groups and Adult Males, indicating this group likely contains both males and females (Fig 3). It’s possible that when the Mid-Size class was detected at the same time as a Social Group, that animal was likely an adult female and when it was detected independently, it could be a juvenile male. Overall, 70% of calculated lengths for the Mid-Size class were greater than the maximum length for females (12 m) [65–67], suggesting that the Mid-Size group could be skewed towards more juvenile males for our study. The seasonal and interannual patterns of the Adult Male and Mid-Size groups show good alignment, further suggesting that the Mid-Size group may consist of more juvenile males rather than large females.

Adult Males were present year-round in the GOA and BSAI, although they were more common in the summer in GOA and fall in BSAI, and less common in the winter and spring (Figs 5–10 and S2–S5 Figs). This seasonal occurrence is consistent with what was previously described from acoustic data in the GOA [18] and at Ocean Station PAPA in the southeast GOA [19]. Whaling data from the northeastern Pacific from 1948 to 1967 also supports this seasonal pattern with an increased mean length of male sperm whales starting between May and June and sustained through September, attributed to the sexual maturity of the animals [3]. Male sperm whales in the GOA are also notorious for longline depredation [70] particularly from the sablefish fishery which has its season from mid-March to mid-November [71], aligning with the peak in presence of Adult Males.

The summer peak in presence and winter low in presence is likely associated with long distance movements of males, between lower latitudes where breeding occurs in the winter/spring and higher latitudes, or feeding grounds, for improved foraging opportunities in the summer/fall [2]. Although this seasonal trend appears to be migratory in nature, there is little evidence that sperm whales have a predictable pattern and/or route to established breeding areas. Rather, they are described as ‘ocean nomads’ based on Discovery Tags used by whalers, that revealed widespread movements between areas of concentration suggesting that their home ranges can span thousands of kilometers [2,4,8,72]. Discovery Tagged animals in southern California and northern Baja California were found in locations ranging from offshore California, Oregon, British Columbia, and the western Gulf of Alaska [8]. More recently, studies using satellite tags have corroborated the nomadic behavior of sperm whales. In a study that tagged 10 sperm whales in the GOA, seven stayed within the GOA, one traveled to British Columbia and back, while three traveled south to the Sea of Cortez, Baja, and offshore Mexico through the California Current without stopping and with no synchronized departure [73]. Although the three southbound whales did all leave before winter when sperm whale presence was at its lowest, there is no evidence that the animals ‘migrate’ to a specific area outside of their home range. There is also photo-identification evidence from the North Atlantic that sperm whales travel from higher latitudes areas like the Azores, to tropical latitudes like the Gulf of Mexico and Bahamas [74] but no concrete evidence that the animals have a pattern or routine to where and when they travel between presumed higher latitude foraging and lower latitude breeding grounds. Instead, it appears that sperm whales travel in response to the distribution of their often-patchy prey sources [4,75] and are linked with temporary breeding sites with favorable prey conditions driven by the effects of oceanographic conditions. It has also been suggested that sexually mature males don’t breed every year and may choose to remain in higher latitudes some years to feed [76] further complicating their seasonal patterns in and out of their home ranges which can span ocean basins. This is also supported by our acoustic observation of year-round presence of Adult Males in the entire study region (Figs 5–8). Historical whaling and satellite tag studies provide evidence of highly variable timing and direction of sperm whale movement but incorporating increased observations over longer timescales is necessary to clarify their behavior as nomadic or migratory. Genetic studies reveal that males in the North Pacific have widespread origin and are likely a mix of males from several independent populations in the Pacific [6] further supporting their nomadic nature. If in fact, sperm whales are truly nomadic animals, understanding how they spatiotemporally exploit available resources is important to establishing management and conservation strategies.

Mid-Size animals were also present year-round in the GOA and BSAI, with a slightly offset peak presence from the Adult Males in both regions (Figs 5–10 and S2–S5 Figs). If we assume that the Mid-Size class is made up of mostly sexually immature males, they likely do not undergo long distance movements to breeding grounds. In the GOA, the peak presence of Mid-Size animals was in the fall or spring months (Fig 9). In the BSAI, peak presence of Mid-Size was in the summer, before the peak presence of Adult Males in the fall, suggesting avoidance of Adult Males by Mid-Size animals (Fig 9). There is evidence of aggression between mature sperm whales based on heavy scarring on their heads [2,77,78]. Some juvenile males may avoid an area during peak presence of mature Adult Males to avoid direct competition, although these groups do overlap on an hourly scale in our data, suggesting temporal overlap of habitat use on some level.

Social Groups were present at all seven recording sites but were not present year-round and instead had distinct seasonal patterns that varied from site to site (Figs 5–10 and S2–S5 Figs). Social Group presence in the winter months between 2010 and 2012 at site BD is consistent with the 2008 sighting of a group of females and immature animals in the Central Aleutians in winter (S2 Fig) [11]. That sighting was considered rare since only males had been observed in ten years of summer sighting surveys previously conducted in the BSAI region [11]. There is also historic whaling evidence that female sperm whales have overwintered in the western Aleutians [8–14]. The continued return of Social Groups to this region in the winter, when productivity is generally lower, could be a sign of site fidelity. Although sperm whales have been described as ‘ocean nomads’, there is evidence from females in the Eastern Caribbean [79,80], North Atlantic [81], western Mediterranean [82], and males in the GOA [73] that site fidelity is a factor in their habitat choice. The presence of Social Groups in certain regions of the BSAI in the winter could be evidence of geographic specializations [80].

In the GOA, Social Group peak presence was in the spring (Fig 9). Seasonal prediction models in the waters of coastal British Columbia found female sperm whales virtually absent after May [3]. The absence of females in the British Columbia model predictions [3] suggests that Social Groups could be traveling further north to the GOA in the spring months. The spring peak was also seen in historical whaling data from the northeastern Pacific where female sperm whales were more often caught from March-May and less often caught from June-September [3]. Previous understanding of female sperm whale distribution post-whaling did not include the GOA.

Contemporary presence of Social Groups in the GOA and BSAI could represent a return to pre-whaling distributions of sperm whales. Females were illegally caught in high numbers in the North Pacific in the 19^th^ and 20^th^ centuries, removing a significant portion of the reproductively mature population [12]. The impacts of whaling on their population, especially given their social ecology, may have been disproportionately large [8,83]. Social Group presence could also represent a change in the distribution of their preferred prey, given how closely sperm whale distribution is linked to squid [75,84]. The BD recording site was located on the nutrient-rich northern side of the Aleutian Islands in the Bering Sea which would be a prime location for squid and provide suitable habitat for sperm whales [85]. Presence of Social Groups could also be related to changes in the water temperature. Nishiwaki (1966) hypothesized that Social Group presence in the BSAI was related to water temperatures above 13°C. And in the GOA, ocean heat content (HC) was the most important sperm whale predictor, with a decrease in HC leading to a decrease in animal presence [62]. However, from 2010 to 2012 when our instruments were recording in the BSAI, this region experienced a multi-year sequential continuation of colder than normal ocean temperatures [86], likely below the 13°C threshold for Social Groups hypothesized by Nishiwaki (1996).

Year-round presence of sperm whales in the GOA and BSAI, especially through the winter, indicates high winter productivity and sustained prey availability [62,87,88]. CB had the highest relative presence of sperm whales, particularly of Mid-Size and Adult Males (Fig 4). This site is located along the continental slope which is popular with males in other regions [3] and this site had a sustained presence of sperm whales, even during years with low overall presence in the GOA [15] likely a result of richer biomass productivity. PT and QN, although relatively offshore, correspond to seamounts which are also important sperm whale habitat in the GOA [15] and several other regions due to their complex seafloor characteristics and water circulation [3,89–91]. There is evidence from whaling data that females were generally found farther from shore in the northeastern Pacific [92] potentially explaining the higher proportion of Social Group presence at sites PT and QN (Fig 4). Social Groups also appeared linked to oceanographic features and were present as far north as the western North Pacific Gyre in the western Aleutian Islands, and the Alaska Gyre and Alaska Current in the Gulf of Alaska [72,93]. In the BSAI, site KS appeared to have the highest relative presence of Mid-Size and Adult Male sperm whales, however, this site has less recording effort than BD and a summer recording effort bias (Fig 4). Regional preference between GOA and BSAI was not significant for any of the size classes, indicating that the two regions are both equally capable of providing suitable foraging conditions.

There were temporal (hourly) and spatial (daily) overlaps between all groups at almost all sites (Fig 4). Temporal and spatial overlap of Adult Males and Social Groups occurred at all sites except AB and could imply that mating is possible in GOA or BSAI (Fig 4). Currently we understand that males travel to tropical latitudes to breed, but there is evidence from whaling data that sperm whales were mating in temperate latitudes off the coast of British Columbia where large bulls were mostly found associated with female schools in April and May (93, Pike et al. 1965 [Unpublished]). There is also evidence that the modal breeding month for sperm whales in the North Pacific was April [94] explaining the peak in presence of Social Groups in GOA in spring. Gregr and Trites (2001) [3] hypothesized that by traveling north into temperate latitudes, Social Groups could improve their encounter rates with more mature males that are ready for breeding. There was more daily, or spatial overlap of Adult Males and Mid-Size and less hourly, or temporal overlap, potentially indicating habitat partitioning or avoidance of sexually mature males by juvenile males (Fig 4).

Interannual variability of sperm whale presence is due to several ecological, behavioral, and environmental factors related to prey availability in the region, namely squid, fish, and skates [95–99]. The observed peak in presence in 2011 in this study aligns with high squid catches [100] and is consistent with observations from southeast GOA (Fig 10) [62]. Density and abundance values from visual surveys in the GOA also support the dip in presence seen in our study in 2013 by Adult Males and Mid-Size, with increasing density and abundance in 2015 (Fig 10) [15]. These dips and peaks in presence are likely a result of changes in prey distribution and abundance which can be difficult to study. Instead, researchers often rely on understanding how small- and large-scale drivers of ocean productivity optimize feeding and spawning conditions of their prey which ultimately impacts aggregation [101,102]. However, the relationship between prey and their environment in the GOA and BSAI, particularly large-scale climate patterns like the PDO, ENSO, and NPGO, is complex and poorly understood. Squid are a highly mobile and adaptable group of marine animals that can be found in a wide range of oceanic conditions driven by prey availability and abundance (zooplankton and forage fish), predator populations (salmon, toothed whales, sablefish, and grenadiers), and changes in habitat quality [100]. In the GOA and BSAI, there are 15 species of squid with unknown abiotic habitat preferences that are likely related to pelagic conditions and currents throughout the North Pacific [100]. A large climate shift in the mid-1970s from a cold to warm regime [103] resulted in a southward shift and intensification of the Aleutian Low pressure system and warmer ocean temperatures [104]. This led to increased zooplankton biomass, demersal, pelagic fish and cephalopod recruitment and abundance [104–106], and declining forage fish populations [107] impacting piscivorous sea birds and some marine mammal populations [106,108,109]. There is also evidence that warmer ocean temperatures and a shoaling of the Oxygen Minimum Zone in the California Current System are resulting in a northward and offshore expansion of some squid species [110–112] creating an environmental refuge in the GOA and BSAI. So, although squid populations might appear to be increasing in warmer ocean temperatures, this increase could be a result of northward range expansion and the impacts on the endemic species are not understood.

In the southeast GOA, peaks in sperm whale acoustic presence seasonally and interannually were related to higher temperatures, a shallow mixed layer, a weaker Alaska Gyre, and enhanced eddy formation [62]. These conditions are also associated with El Niño, (ENSO warm phase), [113,114] which has been shown to be positively correlated with peaks in sperm whale presence up to one year later in the southeast GOA [62] even though ENSO primarily affects lower latitude climates [115]. El Niño conditions during our recording effort persisted in the GOA from 2014 to 2016 and 2018 to 2019 with very strong conditions from 2015 to 2016 (S6–S8 Figs) [116]. Moderate to strong La Niña (ENSO cool phase) conditions were seen from 2010 to 2012 and a weak La Niña from 2016 to 2018 (S6–S8 Figs) [116]. In this study, opposite to what was seen in the southeast GOA, higher monthly sperm whale presence was associated with La Niña conditions, or a negative ONI (S10 Fig). La Niña conditions in the northeastern Pacific are characterized by decreased ocean temperatures, weaker than normal eddies, deeper mixed layer, increased winter nutrient levels, and a return of summer upwelling [117].

Larger scale environmental variability such as the PDO could also influence the presence of sperm whales. Like ENSO, the PDO has a positive, or warm phase, and a negative, or cool phase and these two climate patterns interact with one another. When PDO is highly positive, El Niño will likely be stronger and when the PDO is highly negative, La Niña will likely be stronger [118]. A positive PDO brings environmental conditions that have been connected to increased sperm whale presence in the southeast GOA such as higher temperatures and ocean heat content, a shallow mixed layer, and a weaker Alaska Gyre [103,113,119]. Our study found a significant negative correlation between PDO and sperm whale presence of all classes (Fig 11, S9–S10 Figs and S3 Table). The GLM models with and without lags displayed a significant negative correlation, implying that the effects of the PDO on sperm whale presence span larger time scales (Fig 11, S9 and S10 Figs and S3 Table). At the start of our recording effort in 2011, the PDO was in a cool phase until it flipped to a warm phase in 2014 (S6–S8 Figs) [120]. This PDO inflection point is also reflected in the sperm whale presence where several low presence years following the shift are associated with a very positive PDO phase (S6–S8 Figs). Although the PDO remains in a warm phase for the remainder of our recording effort, the PDO index does decrease dramatically after 2017, and is associated with a steady increase of sperm whale presence through 2019 (S6–S8 Figs). It is important to note that while our nine-year time series likely does include an important phase switch of the PDO in 2014, the PDO cycle occurs at approximately 20-to-30-year time intervals [103] and it is unlikely that our data are sufficient to capture the full relationship between sperm whale presence and the PDO.

Related closely to ENSO and the PDO, the NPGO is a climate pattern that is significantly correlated to fluctuations in salinity, nutrients, and chlorophyll-a in the Gulf of Alaska [63]. Our study found a positive correlation between sperm whale presence and the NPGO index, especially during its positive phases, marked by lower SST, higher salinity, chlorophyll-a, and nutrients (Fig 11, S9 and S10 Figs, S3 Table) [63]. There was no significant correlation between Mid-Size presence and the NPGO index or ONI, implying that juvenile male sperm whales are less linked to ENSO conditions and the NPGO (Fig 11, S9 and S10 Figs, S3 Table). It is important to note that the NPGO is most effective at capturing climate patterns south of 38°N [63].

Overall, higher sperm whale presence was related to large-scale environmental variability associated with cooler ocean temperatures, higher salinity, increased chlorophyll-a, and increased upwelling as seen during La Niña, cool PDO phase, and positive NPGO index. Increased nutrient-rich water and higher productivity from La Niña conditions could sustain higher squid populations, although no direct link has been made in the GOA or BSAI. Findings from this study that is focused on the central GOA and BSAI contradict what was seen at Ocean Station PAPA in the southeast GOA [62], possibly due to different recording efforts or the diminishing effectiveness of PDO and NPGO as predictors of change in marine environments [121]. A study by Litzow et al. 2020 [121] found that since 1988/1989, the main drivers of the PDO and NPGO have become less active, making these large-scale climate patterns less effective at understanding and predicting marine productivity.

From 2014 to 2016, the northeastern Pacific experienced an unprecedented marine heatwave, often referred to as “The Blob” [122], leading to more than 2.5°C increase of the upper 100 m of the ocean [123] resulting in low chlorophyll concentrations that wreaked havoc on marine ecosystems from California to Alaska [124]. Low primary productivity likely resulted in poor foraging conditions revealed by the decrease of Mid-Size and Adult Males and complete absence of Social Groups in the GOA. However, there was no significant relationship between the MHW forecast and sperm whale presence for any of the models or classes (Fig 11, S9 and S10 Figs, S3 Table). The absence of recording effort from this study in 2016 may have hindered capturing the full effects of the marine heatwave in the GOA. Sperm whale presence began to slowly increase in 2015, continuing until the end of our recording effort (S6 and S8 Figs). There appeared to be a large increase in presence of Adult Males and a decrease of Social Groups in 2018 for the All-Site model which is likely influenced by recording effort bias from one site that year (CB) (S8 Fig). Climate models for the North Pacific predict environmental changes that would support higher concentrations of prey and attract top predators like sperm whales in high latitudes [62,111,125]. Although sperm whale presence in this study appears to increase at the end of the recording period in 2018 and 2019, recording effort bias and the lack of consecutive years with increased presence prevents drawing conclusions about the GOA serving as a foraging refuge for the whales (S7 Fig).

The spatiotemporal models in this study only investigated seasonal, interannual trends, and differences in site and region and low model performance was not surprising (S2 Table). The models would be improved with the inclusion of environmental data that is correlated with sperm whale presence such as ocean heat content, sea surface temperature, vertical stratification [62], chlorophyll-a [90,126], mesoscale features like thermal fronts [127] and eddies [90]. This study was also limited by short and/or discontinuous time series at certain sites. Continuation of acoustic monitoring at these sites will allow for more robust time series and potentially improve performance of spatiotemporal models. This is particularly important for the Aleutian Island and two seamount sites where Social Group presence was high, highlighting critical habitat for females and their young in this high latitude region.

## Conclusions

This work highlights the importance of understanding sperm whale spatiotemporal distribution and regional demographics for informing appropriate management and conservation measures. Currently, management of the North Pacific stock of sperm whales does not account for Social Group habitat use and assumes that the region is dominated by juvenile and sexually mature males. This study reveals that Social Group presence in this region is likely overlooked and historical presence of females in whaling data and contemporary ‘rare’ occurrences should not be ignored when determining management practices for this stock. Male and female sperm whales have differences in behavior and ecology that likely translate to demographic specific responses to increasing anthropogenic threats and climate change. Creating a baseline understanding of what Social Group presence looks like in the GOA and BSAI is crucial for monitoring future changes to the demographic composition of the North Pacific stock.

## Supporting information

S1 Fig Interclick interval distribution at each site. Social Groups in green, Mid-Size in orange, and Adult Males in blue. A kernel smoothing function is represented by the bold line outlining the distributions.
(TIFF)

S2 Fig Seasonal trends for the two seamount sites in the gulf of alaska and one island site in the Bering Sea/Aleutian Islands. Seasonality plots for the two seamount sites in the GOA (PT and QN) and one island site in the Bering Sea/Aleutian Islands (BD). Each row represents outputs from the different size class models for each site: a) Inclusive (grey), b) Social Groups (green), c) Mid-Size (orange), and d) Adult Males (blue). Julian day is represented as months as well as by seasons (Wi: December—February, Sp: March—May, Su: June–August, Fa: September–November). The blue histograms at the top denote effort. All plots include 95% confidence intervals represented by the grey shading surrounding the smooth. Covariates that were not retained in the model or not significant are represented with ‘NA’.
(TIF)

S3 Fig Seasonal and interannual trends for site CB. Seasonality plots (left) and presence by year (right) for site CB. Each row represents outputs from the different size class models for each site: a) Inclusive (grey), b) Social Groups (green), c) Mid-Size (orange), and d) Adult Males (blue). Julian day is represented as month as well as by seasons (Wi: December—February, Sp: March—May, Su: June–August, Fa: September–November). Year is a categorical variable displayed as box plots with the first level centered on zero. Covariates that were not retained in the model or not significant are represented with ‘NA’.
(TIF)

S4 Fig Sperm whale presence by site for the Bering Sea/Aleutian Islands. Each row represents outputs from the different size class models for each site: a) Inclusive (grey), b) Social Groups (green), c) Mid-Size (orange), and d) Adult Males (blue). Site is a categorical variable displayed as box plots with the first level centered on zero. Covariates that were not retained in the model or not significant are represented with ‘NA’.
(TIF)

S5 Fig Site Presence for the Gulf of Alaska. Each row represents outputs from the different size class models for each site: a) Inclusive (grey), b) Social Groups (green), c) Mid-Size (orange), and d) Adult Males (blue). Site is a categorical variable displayed as box plots with the first level centered on zero. Covariates that were not retained in the model or not significant are represented with ‘NA’.
(TIF)

S6 Fig Timeseries of climate variability index/probability and sperm whale presence for site CB. Timeseries of climate variability index/probability (PDO, ONI, NPGO, and MHW; left y-axis) and sperm whale presence (black points; right y-axis) for CB. Sperm whale presence for the PDO, ONI, and NPGO were normalized between -1 and 1 to align with the respective climate variability index axis.
(TIF)

S7 Fig Timeseries of climate variability index/probability and sperm whale presence for the Gulf of Alaska. Timeseries of climate variability index/probability (PDO, ONI, NPGO, and MHW; left y-axis) and sperm whale presence (black points; right y-axis) for GOA. Sperm whale presence for the PDO, ONI, and NPGO were normalized between -1 and 1 to align with the respective climate variability index axis.
(TIF)

S8 Fig Timeseries of climate variability index/probability and sperm whale presence for all-sites. Timeseries of climate variability index/probability (PDO, ONI, NPGO, and MHW; left y-axis) and sperm whale presence (black points; right y-axis) for All-Sites. Sperm whale presence for the PDO, ONI, and NPGO were normalized between -1 and 1 to align with the respective climate variability index axis.
(TIF)

S9 Fig Relationship between sperm whale presence and climate variability index/probability for site CB. GLM plots displaying the relationship between mean monthly presence of sperm whales for site CB and the PDO, ONI, and NPGO index. All PDO and NPGO plots represent an eight-month lag, except for the Social Groups which does not include a lag. All ONI plots represent a nine-month lag. Each row (and color) represents outputs from the different size class models for each variable: Inclusive, Social Groups, Mid-Size, and Adult Males. All plots include 95% confidence intervals represented by the grey shading surrounding the linear regression. The regression formula for each model is displayed in the top left-hand corner. Covariates that were not retained in the model or not significant are represented with ‘NA’.
(TIF)

S10 Fig Relationship between sperm whale presence and climate variability index/probability for the Gulf of Alaska. GLM plots displaying the relationship between mean monthly presence of sperm whales for the GOA region and the PDO, ONI, and NPGO index. All PDO and NPGO plots represent an eight-month lag, and all ONI plots represent a nine-month lag. Each row (and color) represents outputs from the different size class models for each variable: Inclusive, Social Groups, Mid-Size, and Adult Males. All plots include 95% confidence intervals represented by the grey shading surrounding the linear regression. The regression formula for each model is displayed in the top left-hand corner. Covariates that were not retained in the model or not significant are represented with ‘NA’.
(TIF)

S1 Table Distribution of 5-minute bins with class presence. Number of 5-minute bins assigned to each class (SG = Social Groups, MS = Mid-Size, AM = Adult Male) for each site, bins that include more than one size class (SG/MS, SG/AM, MS/AM, SG/MS/AM), bins where classification was not possible (NA = No assignment), and total number of 5-minute bins with detections. The percentage in parenthesis represents the proportion of 5-minute bins that fall into each category.
(DOCX)

S2 Table GEE model evaluation summaries. Model evaluation summaries for all site-specific, regional, and All-Site models. The number of one-hour bins with presence are given by the # of Bins. The coefficient of discrimination is given by Tjur’s R^2^. The percent of residuals within the 95% confidence intervals of binned residual plots are given by the % of Residuals.
(DOCX)

S3 Table GLM model summaries. Generalized linear model (GLM) summaries testing the relationship of sperm whale presence and the PDO, ONI, NPGO, and MHW indices for all GAM/GEE models that included year as a variable (i.e., greater than 5 years of data). For each model and class, significance of the model with no lag is denoted with an asterisk (*) in the column ‘Sig’. Significance of a model with a lag is denoted by a value from 8 to 12 representing the number of lags in months in the column ‘Sig’. Respective p-values and R^2^ values for each GLM is denoted for each significant model. Models that were not significant are denoted by ‘NA’. Models where year was not significant in the corresponding GAM/GEE model are *italicized*.
(DOCX)
