# Supplementary figures and images for: Sperm whale demographics in the Gulf of Alaska and Bering Sea/Aleutian Islands: An overlooked female habitat
Source: PLoS One. 2024 Jul 3;19(7):e0285068. doi: 10.1371/journal.pone.0285068 (PMC11221705; doi:10.1371/journal.pone.0285068)

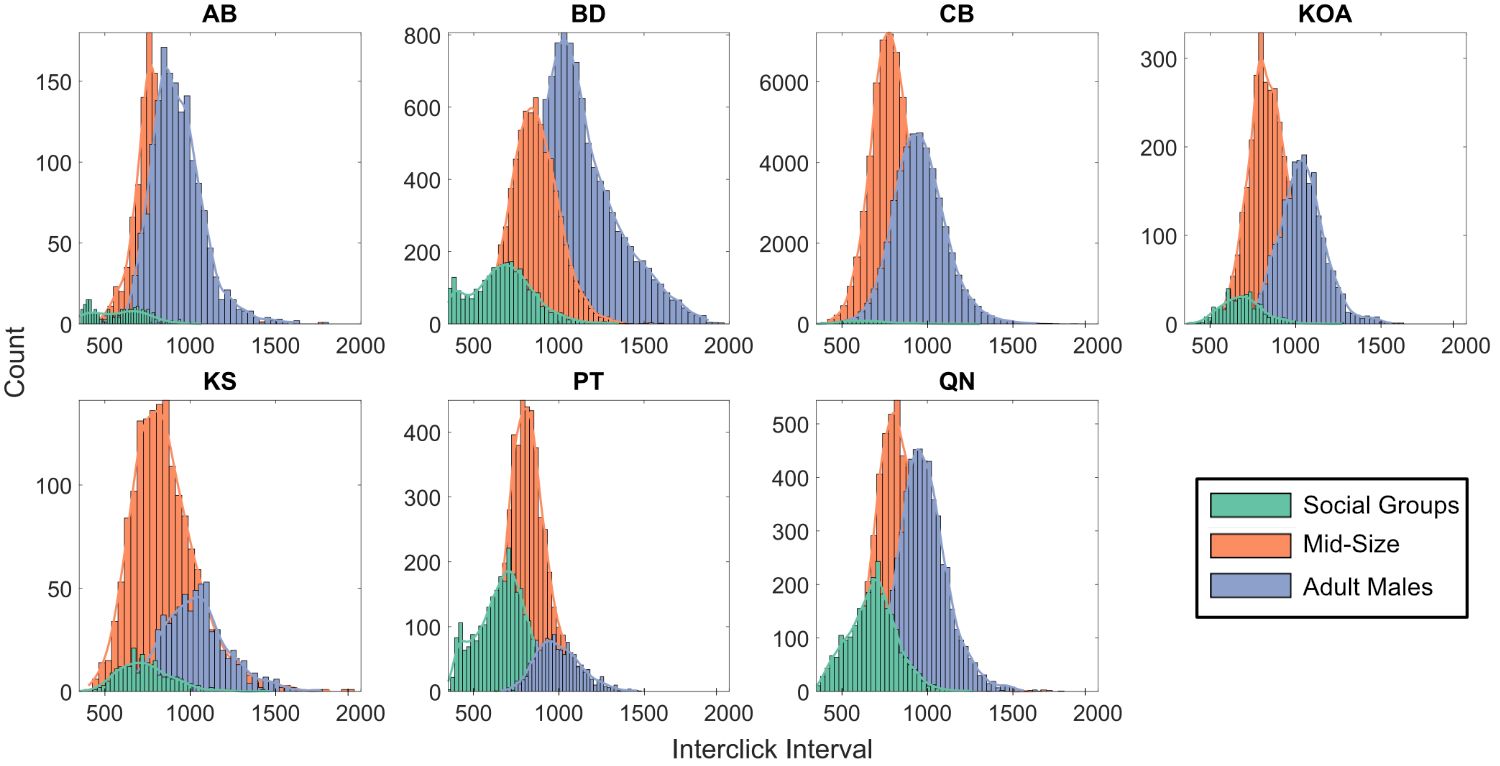

Supplement: S1 Fig — Social Groups in green, Mid-Size in orange, and Adult Males in blue. A kernel smoothing function is represented by the bold line outlining the distributions. (TIFF) [file pone.0285068.s001.tiff]

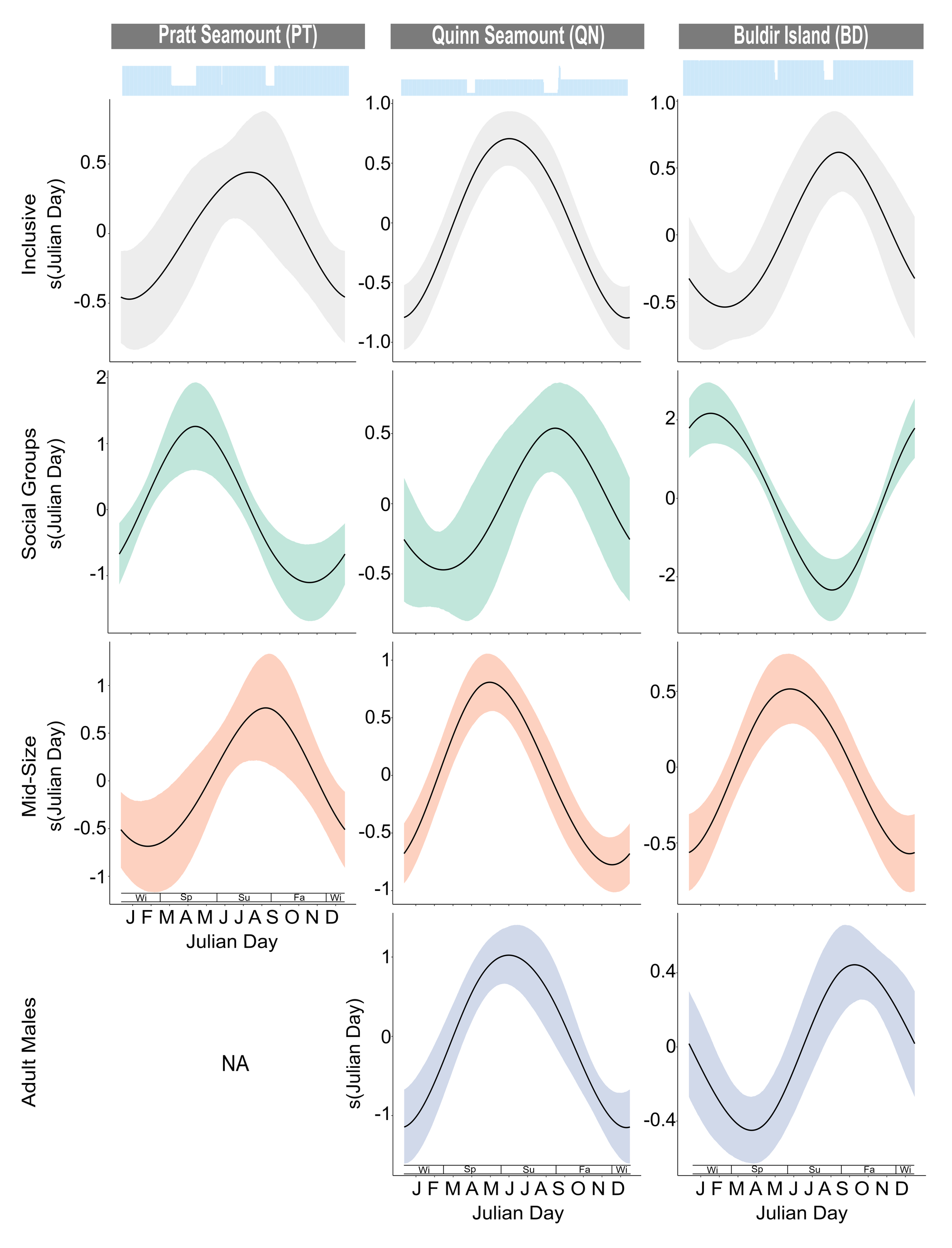

Supplement: S2 Fig — Seasonality plots for the two seamount sites in the GOA (PT and QN) and one island site in the Bering Sea/Aleutian Islands (BD). Each row represents outputs from the different size class models for each site: a) Inclusive (grey), b) Social Groups (green), c) Mid-Size (orange), and d) Adult Males (blue). Julian day is represented as months as well as by seasons (Wi: December—February, Sp: March—May, Su: June–August, Fa: September–November). The blue histograms at the top denote effort. All plots include 95% confidence intervals represented by the grey shading surrounding the smooth. Covariates that were not retained in the model or not significant are represented with ‘NA’. (TIF) [file pone.0285068.s002.tif]

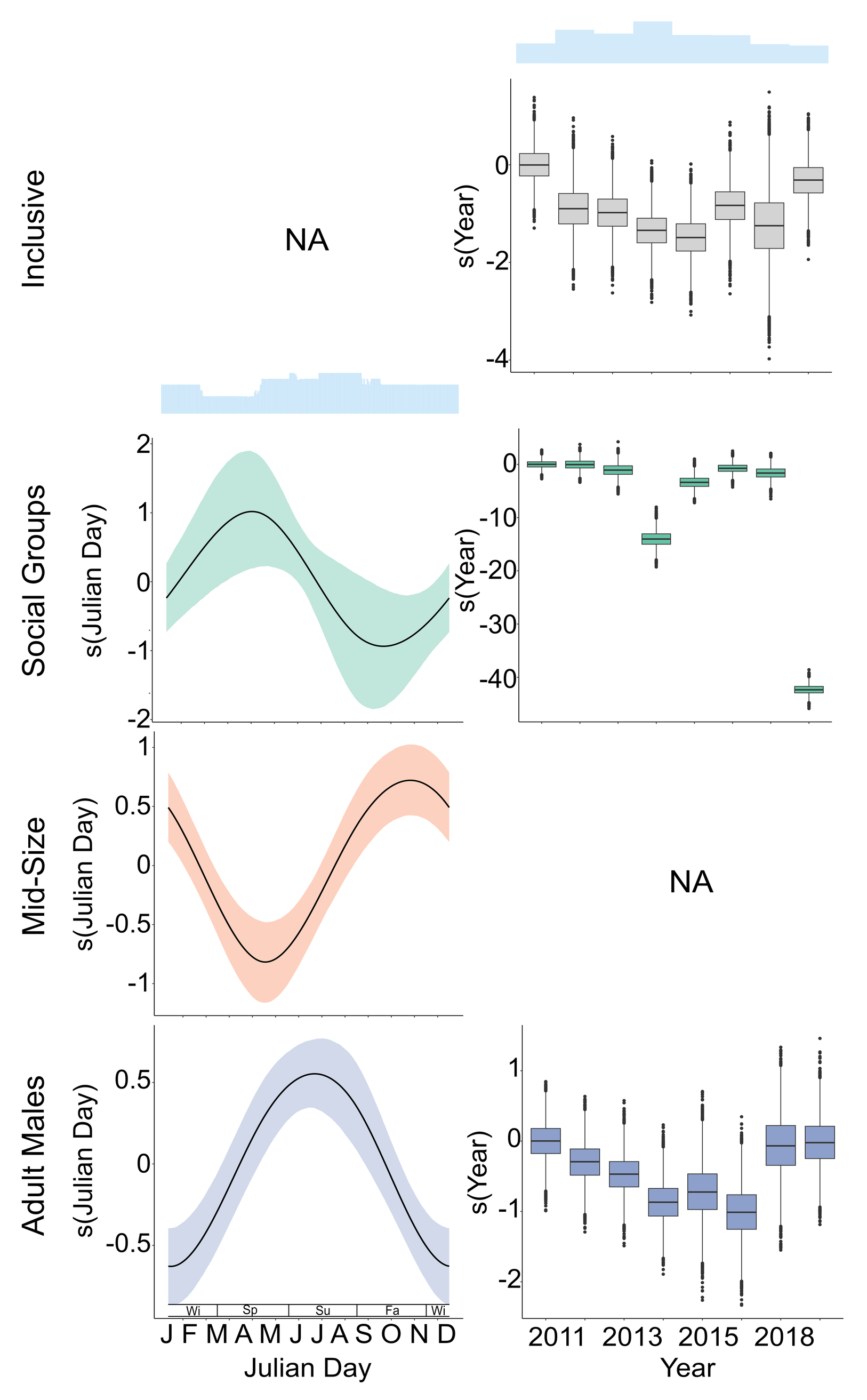

Supplement: S3 Fig — Seasonality plots (left) and presence by year (right) for site CB. Each row represents outputs from the different size class models for each site: a) Inclusive (grey), b) Social Groups (green), c) Mid-Size (orange), and d) Adult Males (blue). Julian day is represented as month as well as by seasons (Wi: December—February, Sp: March—May, Su: June–August, Fa: September–November). Year is a categorical variable displayed as box plots with the first level centered on zero. Covariates that were not retained in the model or not significant are represented with ‘NA’. (TIF) [file pone.0285068.s003.tif]

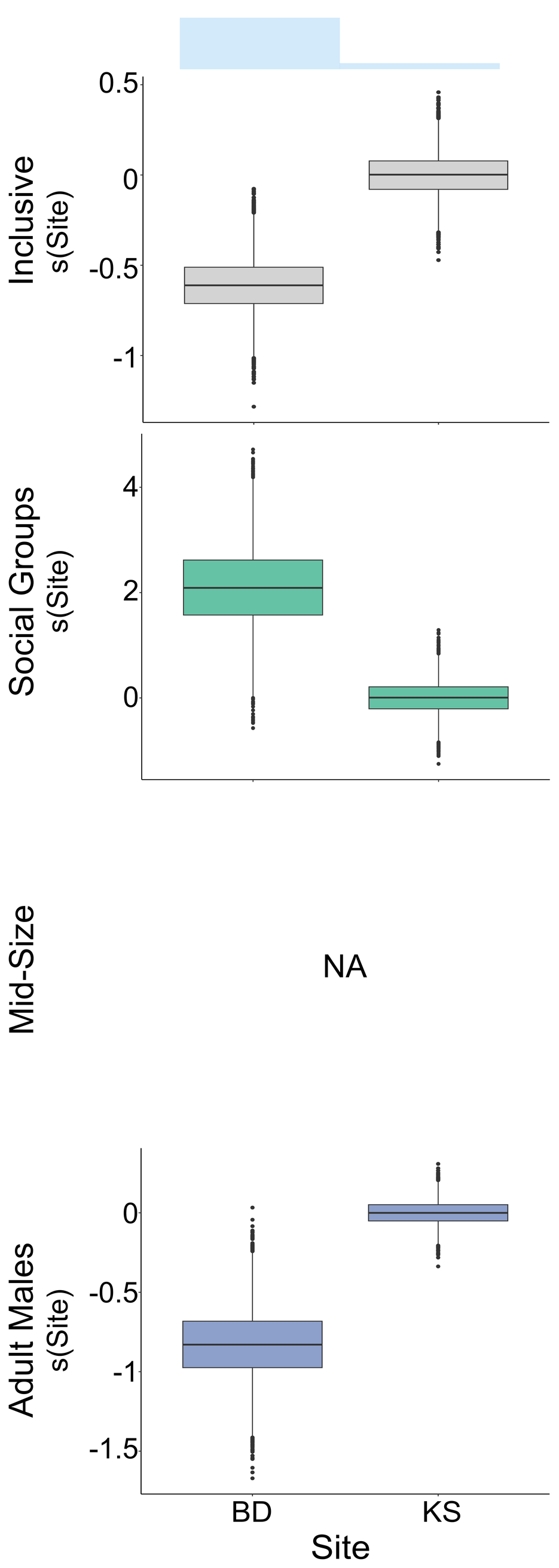

Supplement: S4 Fig — Each row represents outputs from the different size class models for each site: a) Inclusive (grey), b) Social Groups (green), c) Mid-Size (orange), and d) Adult Males (blue). Site is a categorical variable displayed as box plots with the first level centered on zero. Covariates that were not retained in the model or not significant are represented with ‘NA’. (TIF) [file pone.0285068.s004.tif]

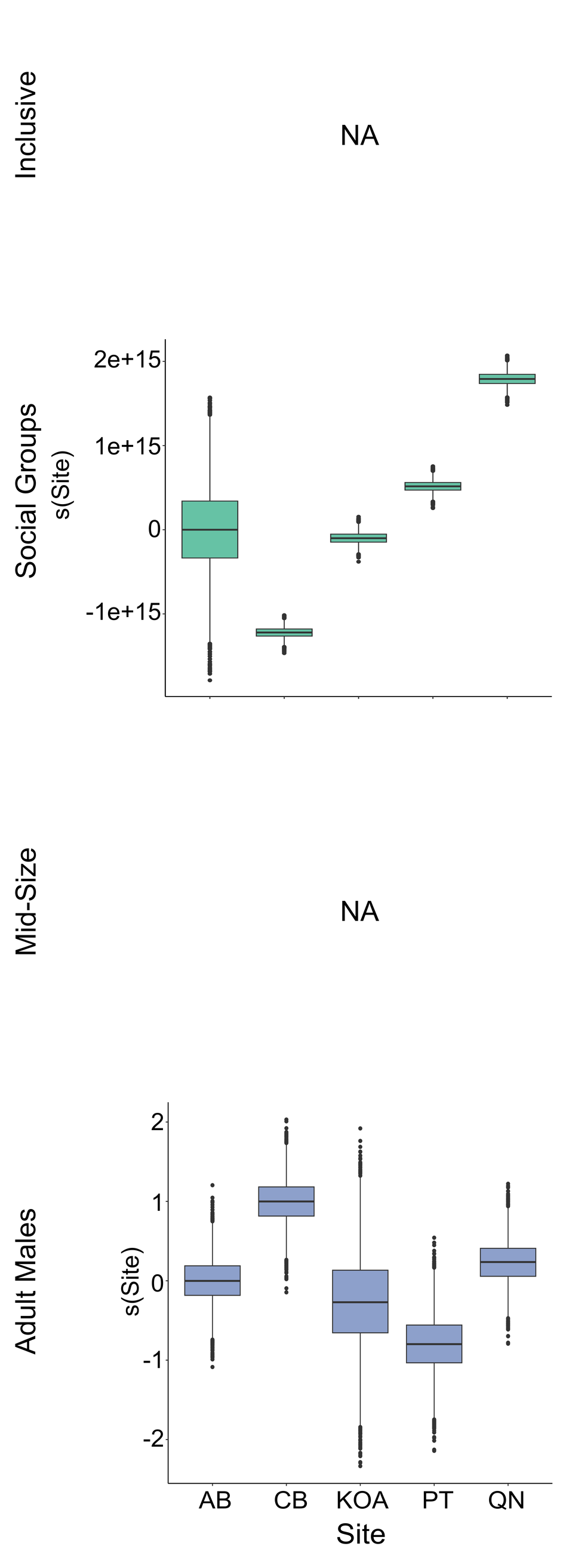

Supplement: S5 Fig — Each row represents outputs from the different size class models for each site: a) Inclusive (grey), b) Social Groups (green), c) Mid-Size (orange), and d) Adult Males (blue). Site is a categorical variable displayed as box plots with the first level centered on zero. Covariates that were not retained in the model or not significant are represented with ‘NA’. (TIF) [file pone.0285068.s005.tif]

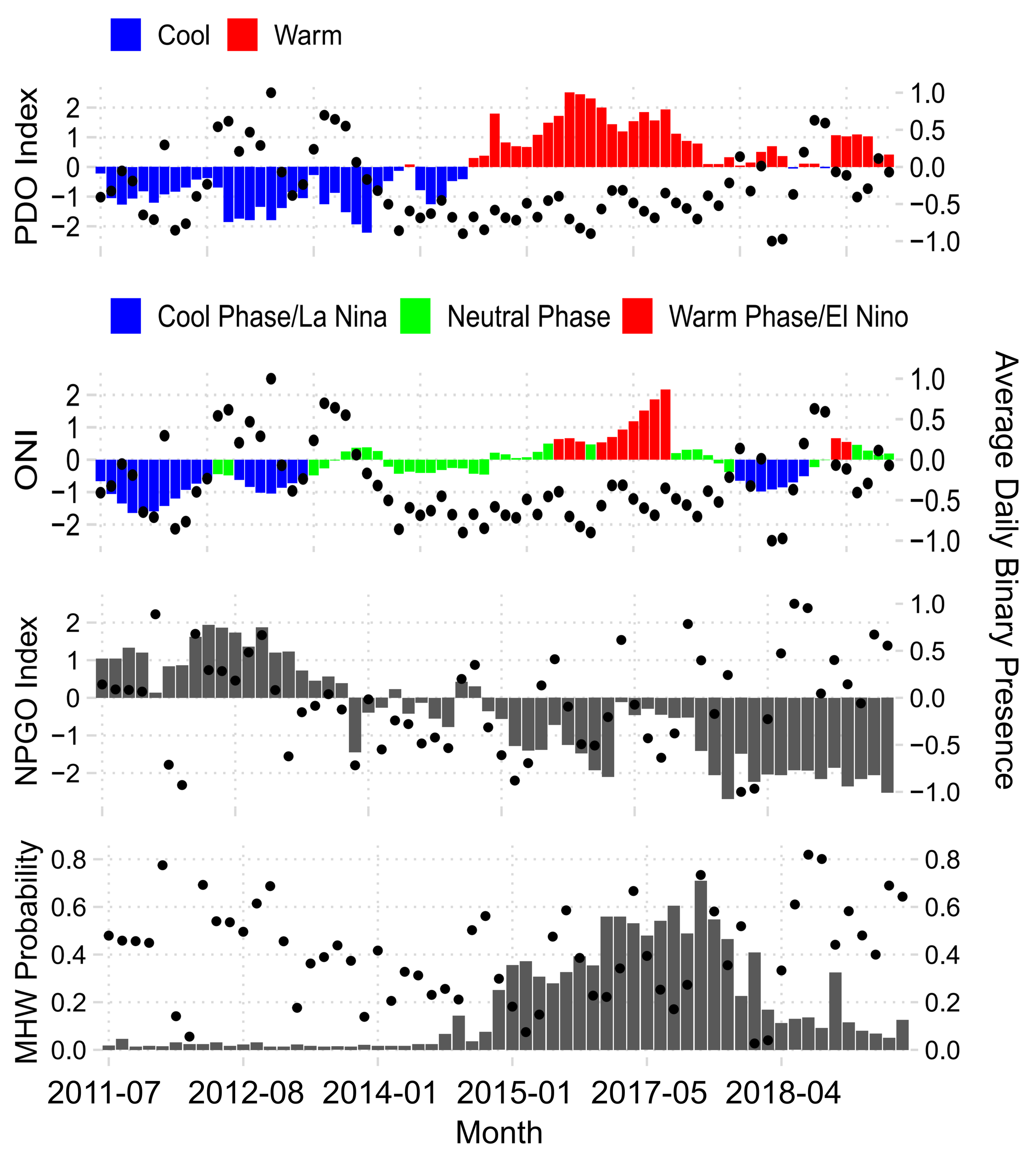

Supplement: S6 Fig — Timeseries of climate variability index/probability (PDO, ONI, NPGO, and MHW; left y-axis) and sperm whale presence (black points; right y-axis) for CB. Sperm whale presence for the PDO, ONI, and NPGO were normalized between -1 and 1 to align with the respective climate variability index axis. (TIF) [file pone.0285068.s006.tif]

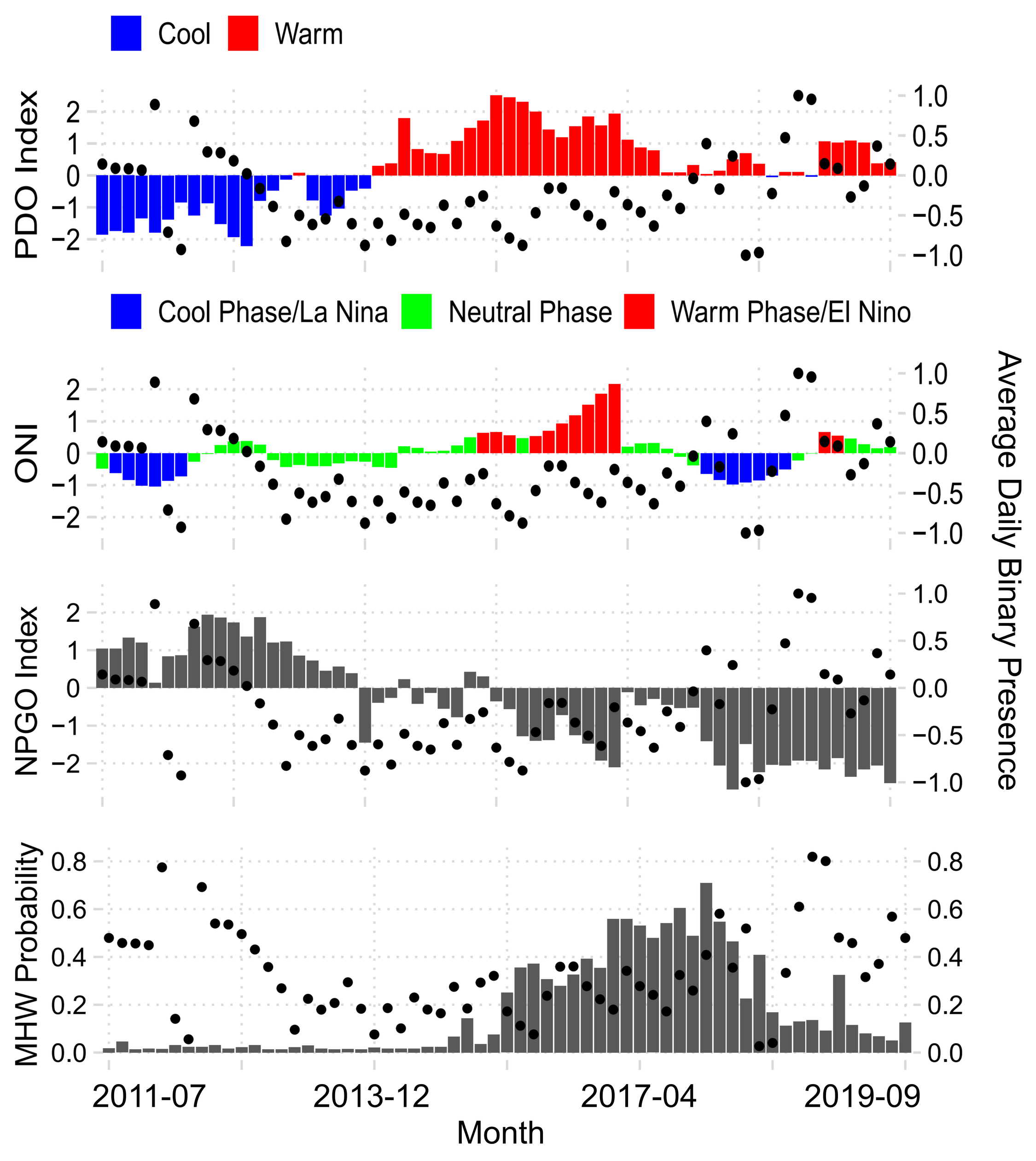

Supplement: S7 Fig — Timeseries of climate variability index/probability (PDO, ONI, NPGO, and MHW; left y-axis) and sperm whale presence (black points; right y-axis) for GOA. Sperm whale presence for the PDO, ONI, and NPGO were normalized between -1 and 1 to align with the respective climate variability index axis. (TIF) [file pone.0285068.s007.tif]

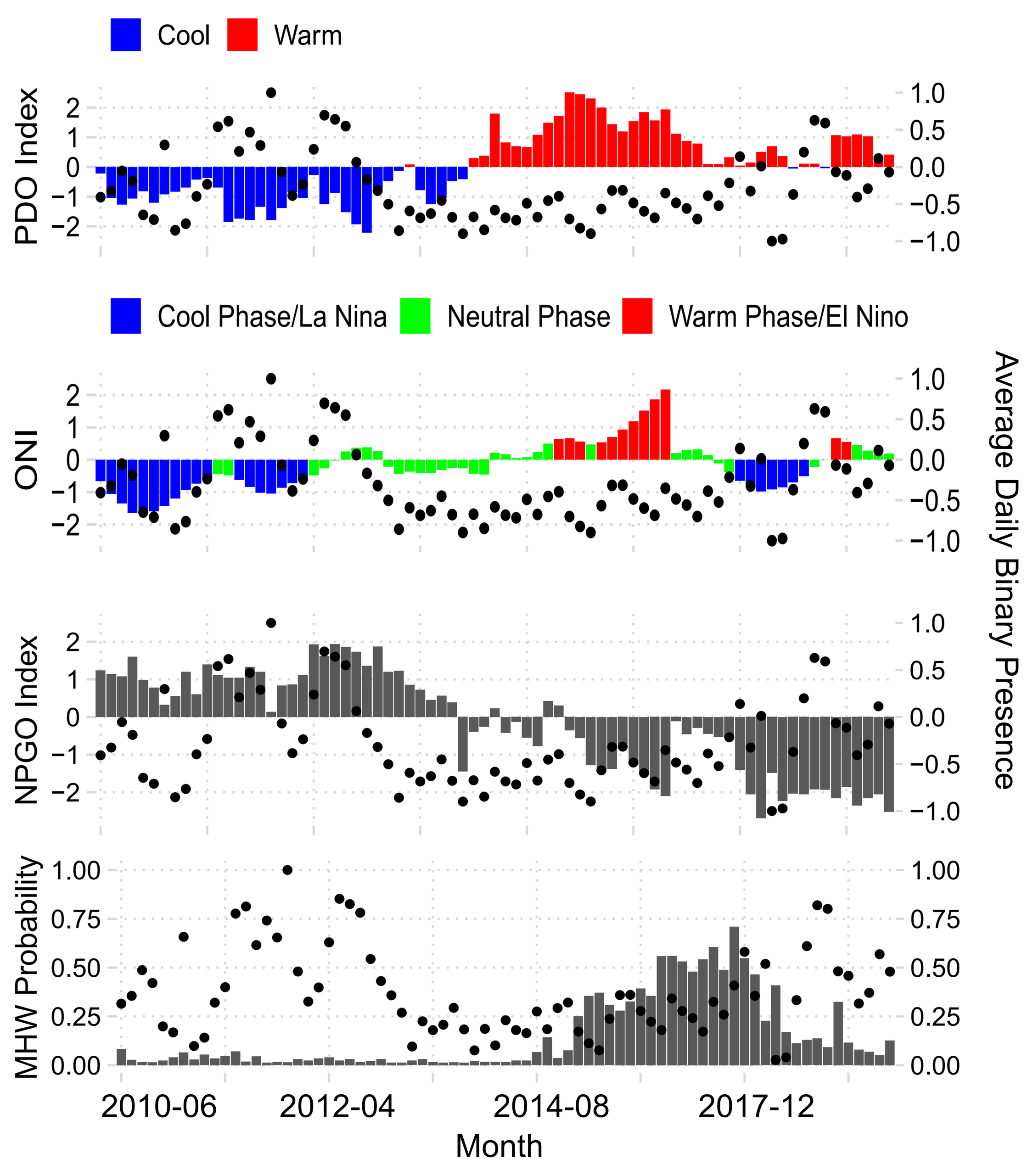

Supplement: S8 Fig — Timeseries of climate variability index/probability (PDO, ONI, NPGO, and MHW; left y-axis) and sperm whale presence (black points; right y-axis) for All-Sites. Sperm whale presence for the PDO, ONI, and NPGO were normalized between -1 and 1 to align with the respective climate variability index axis. (TIF) [file pone.0285068.s008.tif]

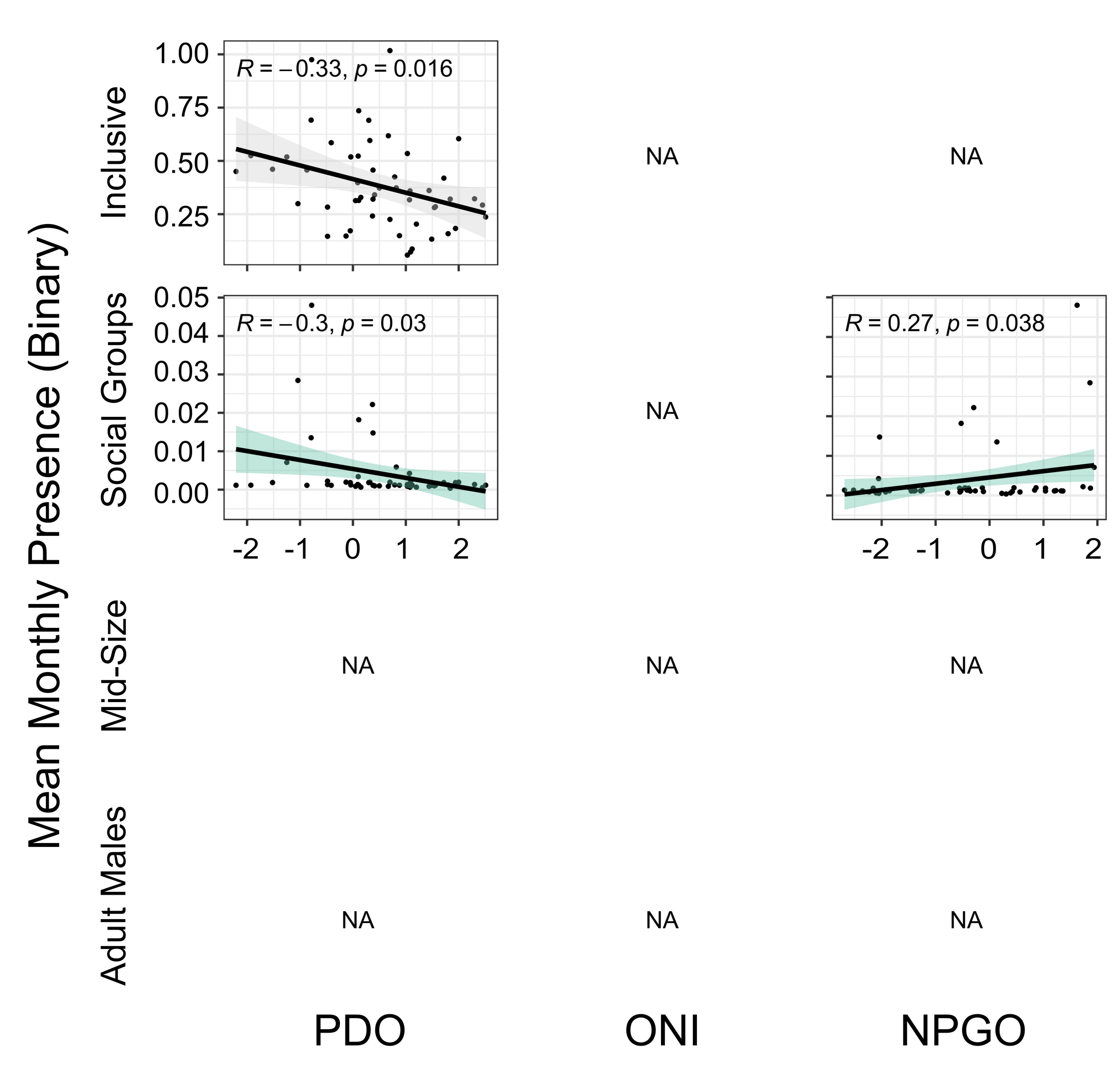

Supplement: S9 Fig — GLM plots displaying the relationship between mean monthly presence of sperm whales for site CB and the PDO, ONI, and NPGO index. All PDO and NPGO plots represent an eight-month lag, except for the Social Groups which does not include a lag. All ONI plots represent a nine-month lag. Each row (and color) represents outputs from the different size class models for each variable: Inclusive, Social Groups, Mid-Size, and Adult Males. All plots include 95% confidence intervals represented by the grey shading surrounding the linear regression. The regression formula for each model is displayed in the top left-hand corner. Covariates that were not retained in the model or not significant are represented with ‘NA’. (TIF) [file pone.0285068.s009.tif]

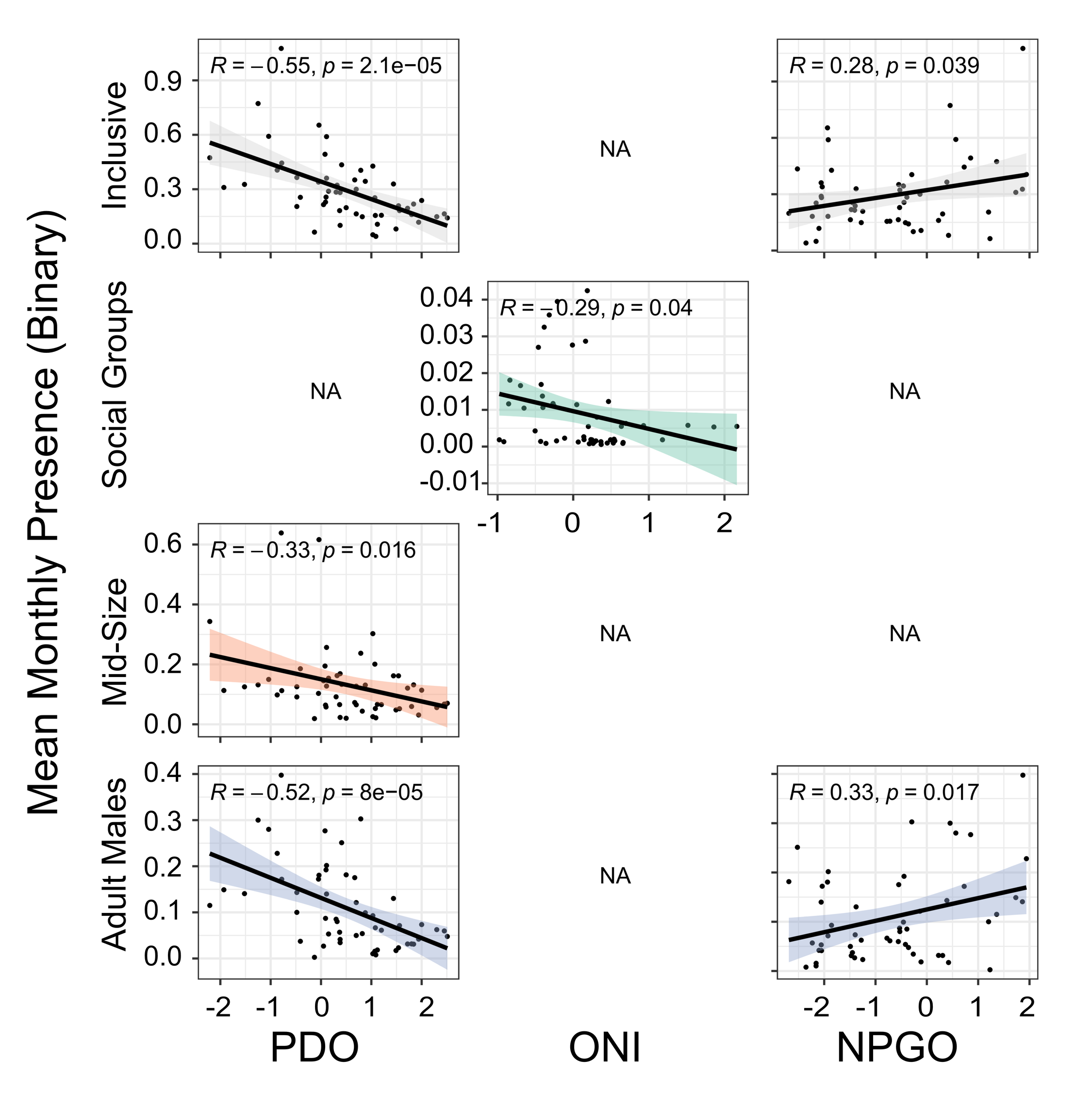

Supplement: S10 Fig — GLM plots displaying the relationship between mean monthly presence of sperm whales for the GOA region and the PDO, ONI, and NPGO index. All PDO and NPGO plots represent an eight-month lag, and all ONI plots represent a nine-month lag. Each row (and color) represents outputs from the different size class models for each variable: Inclusive, Social Groups, Mid-Size, and Adult Males. All plots include 95% confidence intervals represented by the grey shading surrounding the linear regression. The regression formula for each model is displayed in the top left-hand corner. Covariates that were not retained in the model or not significant are represented with ‘NA’. (TIF) [file pone.0285068.s010.tif]
