# Supplementary material for: Sperm whale demographics in the Gulf of Alaska and Bering Sea/Aleutian Islands: An overlooked female habitat
Source: PLoS One. 2024 Jul 3;19(7):e0285068. doi: 10.1371/journal.pone.0285068 (PMC11221705; doi:10.1371/journal.pone.0285068)
Supplement: S1 Table — Number of 5-minute bins assigned to each class (SG = Social Groups, MS = Mid-Size, AM = Adult Male) for each site, bins that include more than one size class (SG/MS, SG/AM, MS/AM, SG/MS/AM), bins where classification was not possible (NA = No assignment), and total number of 5-minute bins with detections. The percentage in parenthesis represents the proportion of 5-minute bins that fall into each category. (DOCX) [file pone.0285068.s011.docx]

Table S1. Number of five-minute bins assigned to each class (SG = Social Groups, MS = Mid-Size, AM = Adult Male) for each site, bins that include more than one size class (SG/MS, SG/AM, MS/AM, SG/MS/AM), bins where classification was not possible (NA = No assignment), and total number of 5-minute bins with detections. The percentage in parenthesis represents the proportion of five-minute bins that fall into each category rounded to one decimal place.

| **Site** | **SG** | **MS** | **AM** | **SG/MS** | **SG/AM** | **MS/AM** | **SG/MS**  **/AM** | **NA** | **Total Bins Detected** |
| --- | --- | --- | --- | --- | --- | --- | --- | --- | --- |
| **CB** | 1114  (1%) | 55857  (49.1%) | 45088  (39.6%) | 37  (0%) | 23  (0%) | 596  (0.5%) | 3  (0%) | 11005  (9.7%) | 113723 |
| **PT** | 2754  (30.5%) | 4503  (49.9%) | 1211  (13.4%) | 30  (0.3%) | 2  (0%) | 58  (0.6%) | 2  (0%) | 461  (5.1%) | 9021 |
| **QN** | 2521  (18.4%) | 4646  (33.9%) | 5433  (39.6%) | 0  (0%) | 2  (0%) | 7  (0.1%) | 0  (0%) | 1103  (8%) | 13712 |
| **AB** | 277  (6.8%) | 1257  (37.4%) | 1597  (47.5%) | 0  (0%) | 0  (0%) | 2  (0.1%) | 0  (0%) | 277  (8.2%) | 3360 |
| **KOA** | 388  (6.1%) | 2977  (46.7%) | 1961  (30.8%) | 39  (0.6%) | 11  (0.2%) | 66  (1%) | 0  (0%) | 927  (14.6%) | 6369 |
| **BD** | 2699  (9.3%) | 6623  (22.8%) | 9699  (33.4%) | 49  (0.2%) | 23  (0.1%) | 259  (0.9%) | 0  (0%) | 9662  (33.3%) | 29014 |
| **KS** | 212  (7%) | 1603  (52.6%) | 665  (21.8%) | 3  (0.1%) | 0  (0%) | 1  (0%) | 0  (0%) | 563  (18.5%) | 3047 |
