# Supplementary material for: Sperm whale demographics in the Gulf of Alaska and Bering Sea/Aleutian Islands: An overlooked female habitat
Source: PLoS One. 2024 Jul 3;19(7):e0285068. doi: 10.1371/journal.pone.0285068 (PMC11221705; doi:10.1371/journal.pone.0285068)
Supplement: S2 Table — Model evaluation summaries for all site-specific, regional, and All-Site models. The number of one-hour bins with presence are given by the # of Bins. The coefficient of discrimination is given by Tjur’s R2. The percent of residuals within the 95% confidence intervals of binned residual plots are given by the % of Residuals. (DOCX) [file pone.0285068.s012.docx]

| Model | Site/Region | Sex | # of Bins | Tjur’s R^2^ | % of Residuals |
| --- | --- | --- | --- | --- | --- |
| Site | BD | Inclusive | 8674 | 0.038 | 36% |
|  |  | Social Groups | 533 | 0.044 | 20% |
|  |  | Mid-Size | 2331 | 0.018 | 55% |
|  |  | Adult Males | 2547 | 0.012 | 50% |
|  | PT | Inclusive | 1862 | 0.010 | 43% |
|  |  | Social Groups | 361 | 0.020 | 11% |
|  |  | Mid-Size | 887 | 0.014 | 34% |
|  |  | Adult Males | 277 | 0.003 | 32% |
|  | QN | Inclusive | 3215 | 0.039 | 46% |
|  |  | Social Groups | 377 | 0.002 | 25% |
|  |  | Mid-Size | 954 | 0.014 | 38% |
|  |  | Adult Males | 874 | 0.023 | 48% |
|  | CB | Inclusive | 20567 | 0.039 | 50% |
|  |  | Social Groups | 178 | 0.007 | 7% |
|  |  | Mid-Size | 9389 | 0.045 | 30% |
|  |  | Adult Males | 7419 | 0.041 | 48% |
| Region | BSAI | Inclusive | 9600 | 0.036 | 42% |
|  |  | Social Groups | 581 | 0.038 | 23% |
|  |  | Mid-Size | 2797 | 0.021 | 54% |
|  |  | Adult Males | 2700 | 0.015 | 49% |
|  | GOA | Inclusive | 27749 | 0.091 | 33% |
|  |  | Social Groups | 1028 | 0.007 | 25% |
|  |  | Mid-Size | 12092 | 0.038 | 38% |
|  |  | Adult Males | 9227 | 0.049 | 43% |
| All-Site | | Inclusive | 37349 | 0.077 | 35% |
|  |  | Social Groups | 1609 | 0.010 | 30% |
|  |  | Mid-Size | 14889 | 0.019 | 42% |
|  |  | Adult Males | 11927 | 0.034 | 48% |
