# Supplementary material for: Sperm whale demographics in the Gulf of Alaska and Bering Sea/Aleutian Islands: An overlooked female habitat
Source: PLoS One. 2024 Jul 3;19(7):e0285068. doi: 10.1371/journal.pone.0285068 (PMC11221705; doi:10.1371/journal.pone.0285068)
Supplement: S3 Table — Generalized linear model (GLM) summaries testing the relationship of sperm whale presence and the PDO, ONI, NPGO, and MHW indices for all GAM/GEE models that included year as a variable (i.e., greater than 5 years of data). For each model and class, significance of the model with no lag is denoted with an asterisk (*) in the column ‘Sig’. Significance of a model with a lag is denoted by a value from 8 to 12 representing the number of lags in months in the column ‘Sig’. Respective p-values and R2 values for each GLM is denoted for each significant model. Models that were not significant are denoted by ‘NA’. Models where year was not significant in the corresponding GAM/GEE model are italicized. (DOCX) [file pone.0285068.s013.docx]

|  | **Class** | **PDO** | | | **ONI** | | | | | **NPGO** | | | **MHW** |
| --- | --- | --- | --- | --- | --- | --- | --- | --- | --- | --- | --- | --- | --- |
|  |  | **Sig** | **P-Value** | **R^2^** | **Sig** | **P-Value** | | **R^2^** | | **Sig** | **P-Value** | **R^2^** |  |
| **CB** | Inclusive | *  8 | 0.01  0.0016 | -0.33  -0.33 | NA | | | | | NA | | | NA |
|  | Social Groups | *  8  9  10 | 0.045  0.03  0.012 0.047 | -0.26  -0.3  -0.35  -0.28 | NA | | | | | * | 0.038 | 0.27 | NA |
|  | Mid-Size | *NA* | | | *NA* | | | | | *NA* | | | *NA* |
|  | Adult Males | NA | | | NA | | | | | NA | | | NA |
| **GOA** | Inclusive | *  8  9  10 | 0.007 2.1e-05 0.00023 0.01 | -0.34  -0.55  -0.49  -0.36 | NA | | | | | 8 | 0.039 | 0.28 | NA |
|  | Social Groups | NA | | | 9  10 | | 0.04  0.049 | | -0.29  -0.28 | NA | | | NA |
|  | Mid-Size | 8 | 0.016 | -0.33 | NA | | | | | NA | | | NA |
|  | Adult Males | ***  *8*  *9*  *10*  *11*  *12* | *0.025*  *8e-05*  *2.3e-05*  *0.00051*  *0.0034*  *0.035* | *-0.29*  *-0.52*  *-0.55*  *-0.47*  *-0.41*  *-0.3* | *9*  *10* | *0.0396, 0.0449* | | *0.08196, 0.0796* | | *8*  *9*  *10* | *0.017*  *0.0077*  *0.014* | *0.33*  *0.37*  *0.34* | *NA* |
| **All-Site** | Inclusive | *  8  9  10  11  12 | 3e-05  1.1e-06  7.6e-07  2.4e-05  7.9e-05  8e-04 | -0.46  -0.55  -0.57  -0.5  -0.47  -0.41 | NA | | | | | 8  9  10  11  12 | 1.6e-05  4e-05  0.00012  0.0014  0.0038 | 0.5  0.48  0.46  0.39  0.36 | NA |
|  | Social Groups | *  8  9  10  11  12 | 0.023  0.0022  0.012  0.015  0.047  0.015 | -0.26  -0.37  -0.31  -0.3  -0.25  -0.3 | 9  10  11  12 | 0.027  0.007  0.012  0.016 | | -0.27  -0.33  -0.31  -0.3 | | 8  9  10  1112 | 0.0049  0.037  0.0095  0.013  0.01 | 0.34  0.26  0.32  0.31  0.32 | NA |
|  | Mid-Size | *  8  9  10  12 | 0.014  0.0045  0.007  0.038  0.047 | -0.28  -0.34  -0.33  -0.26  -0.25 | NA | | | | | 8 | 0.03 | 0.27 | NA |
|  | Adult Males | *  8  9  10  11  12 | 0.0053  9.7e-06  8.1e-07  1e-05  0.00021  0.02 | -0.32  -0.51  -0.56  -0.52  -0.45  -0.29 | 8  9 | 0.021  0.047 | | -0.28  -0.25 | | 8  9  101112 | 0.0018  3e-04  0.0028  0.0076  0.032 | 0.38  0.43  0.36  0.33  0.27 | NA |
